# Clay mineral formation under oxidized conditions and implications for paleoenvironments and organic preservation on Mars

**DOI:** 10.1038/s41467-017-01235-7

**Published:** 2017-11-01

**Authors:** Seth R. Gainey, Elisabeth M. Hausrath, Christopher T. Adcock, Oliver Tschauner, Joel A. Hurowitz, Bethany L. Ehlmann, Yuming Xiao, Courtney L. Bartlett

**Affiliations:** 1 Department of Geoscience, University of Nevada, Las Vegas, 4505 S. Maryland Pkwy., Las Vegas, NV 89154 USA; 2 Department of Geosciences, State University of New York, Stony Brook, NY 11794-2100 USA; 3 Division of Geological and Planetary Sciences, California Institute of Technology, Pasadena, CA 91125 USA; 4 Jet Propulsion Laboratory, California Institute of Technology, Pasadena, CA 91109 USA; 5 HPCAT, Carnegie Institution of Washington, Argonne, IL 60439 USA

## Abstract

Clay mineral-bearing locations have been targeted for martian exploration as potentially habitable environments and as possible repositories for the preservation of organic matter. Although organic matter has been detected at Gale Crater, Mars, its concentrations are lower than expected from meteoritic and indigenous igneous and hydrothermal reduced carbon. We conducted synthesis experiments motivated by the hypothesis that some clay mineral formation may have occurred under oxidized conditions conducive to the destruction of organics. Previous work has suggested that anoxic and/or reducing conditions are needed to synthesize the Fe-rich clay mineral nontronite at low temperatures. In contrast, our experiments demonstrated the rapid formation of Fe-rich clay minerals of variable crystallinity from aqueous Fe^3+^ with small amounts of aqueous Mg^2+^. Our results suggest that Fe-rich clay minerals such as nontronite can form rapidly under oxidized conditions, which could help explain low concentrations of organics within some smectite-containing rocks or sediments on Mars.

## Abstract (web summary)

In the Gale Crater on Mars, organic matter has been detected, but in much lower concentrations than expected. Here, the authors conduct clay mineral synthesis experiments which suggest that clay minerals may rapidly form under oxidized conditions and thus explain the low organic concentrations in Gale Crater.

## Introduction

Thousands of locations on Mars have rock units containing Fe/Mg-rich clay minerals^1–6^, which can both indicate the past long-term presence of liquid water important for potentially habitable environments, and also preserve organic matter adsorbed onto clay mineral surfaces and interlayers. A key question is the conditions under which these martian clay minerals formed, including both pH and oxidation state. Reducing and/or anoxic conditions might imply subsurface formation, buffered by a basaltic reservoir, or surface conditions in which the atmospheric composition was different than at present. Oxidized conditions would almost certainly be at or near the surface, made oxic by photochemistry and/or atmospheric escape. The precise details of clay mineral composition can serve as fingerprints to constrain the past environmental conditions under which they formed^6^. Previous work by Harder^7^, Decarreau et al.^8^, and Mizutani et al.^9^ has shown that the precipitation of Fe-bearing clay minerals, even the dioctahedral Fe^3+^-rich smectite endmember, nontronite, occurs in solutions containing Fe^2+^ under conditions that are anoxic and/or reducing (Eh < ~−0.2 V)^7^. The initially formed Fe^2+^-bearing precipitates are subsequently oxidized to form nontronite^7,9^. The apparent requirement for initially anoxic and/or reducing conditions is because a divalent cation, e.g., Fe^2+^, is required to stabilize the octahedral layers and promote bi-dimensional (e.g., the *a* and *b* axis) growth of these clay minerals^7,10,11^. Low temperature experimental attempts to synthesize clay minerals from Fe^3+^-bearing solutions without a divalent cation have formed amorphous^7^ and/or very poorly crystalline products^12^.

Here we test whether Fe/Mg clay minerals can form under oxidized conditions at temperatures in which terrestrial life may survive by performing clay mineral synthesis experiments. These experiments were designed to be relevant to potentially habitable environments on Mars, containing a range of chemical compositions, including both Fe and Mg end-members as well as intermediate compositions. We demonstrate the rapid formation of Fe-rich clay minerals of variable crystallinity from aqueous Fe^3+^ in the presence of at least small amounts of Mg^2+^. Our results suggest that Fe^3+^ clay mineral-bearing terrains may not necessarily be conducive to the preservation of organic matter on Mars.

## Results

### XRD and µXRD analyses

Fe-rich clay minerals, including nontronite^8,9^ were rapidly synthesized within 2 days at 150 °C, following 1 day at room temperature and within 60 days at 100 °C following 1 day at room temperature from oxidized, Fe^2+^-free solutions, which contained at least 5% Mg (i.e., a molar ratio of Mg:Fe^3+^ greater than or equal to 1:19 in the solution) (Table 1).

**Table 1:** Chemistry used in selected synthesis experiments^a^

	100-Fe Control^b^	100-Fe^3+^	5-Mg 95-Fe^3+^	15-Mg 85-Fe^3+^	50-Mg 50-Fe^3+^	100-Mg
Chemical Name	Amount Cation (mol)	Amount Cation (mol)	Amount Cation (mol)	Amount Cation (mol)	Amount Cation (mol)	Amount Cation (mol)
Sodium metasilicate—pentahydrate	0.0204	0.0204	0.0204	0.0205	0.0204	0.0205
Iron (II) sulfate—heptahydrate	0.0142	—	—	—	–	–
Sodium dithionite	0.0241	—	—	—	–	–
Iron (III) sulfate—rhomboclase & ferricopiapite	—	0.0138^c^	0.0146^c^	0.0117^c^	0.0069 ^c^	–
Magnesium sulfate—epsomite	—	—	0.0007	0.0022	0.0071	0.0142
Fe:Mg ratio	1:0	1:0	19:1	17:3	1:1	0:1

Solution name	Amount (mL)	Amount (mL)	Amount (mL)	Amount (mL)	**Amount (mL)**	**Amount (mL)**
Sodium hydroxide (5 M)	19.80	19.80	19.80	19.80	19.80	19.80
H_2_O	417.52	419.96	417.11	416.41	417.11	417.87
Sulfuric acid (0.5 M)	41.00	42.00	42.00	42.00	42.00	42.00
Ending pH	12.49	12.69	12.64	12.76	12.79	12.88

All experiments were aged for 1 day at room temperature and then heated for an additional 2 days at 150 °C—for experiments incubated at 100 °C for 60 days see methods

^a^For all experimental conditions see Supplementary Tables 1 and 2

^b^100-Fe bearing control condition, which was synthesized from initially ferrous Fe-containing solutions (sodium dithionite was added to maintain reducing conditions for the ferrous Fe) and then oxidized using published methods^9^

^c^Fe(III) molar concentrations were determined by atomic absorption spectroscopy

**Fig. 1:**
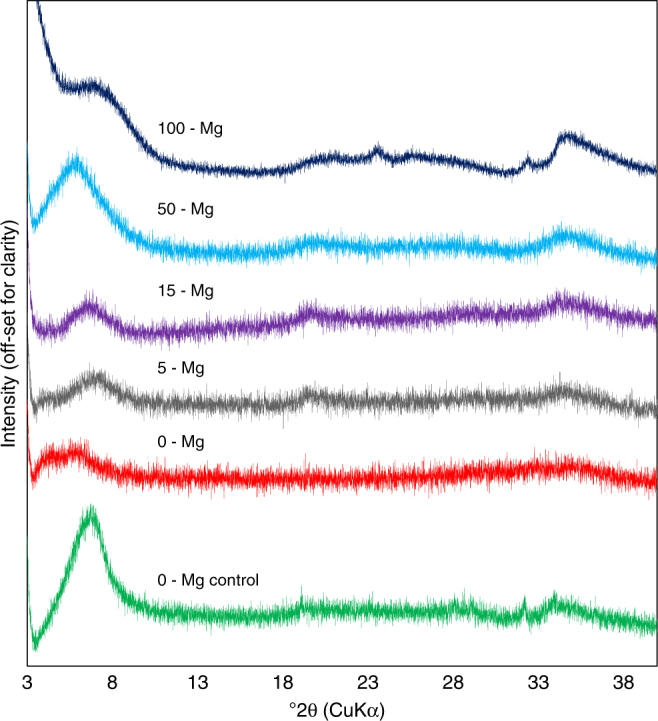
X-Ray diffractograms of the synthetic clay minerals. Samples were analyzed as oriented samples on Si-mounts. Note that the 001 diffraction of the 0-Mg nontronite control, using initial Fe^2+^, indicates that the control is crystalline, whereas the 0-Mg experiment, including only Fe^3+^ is largely amorphous. This is also shown in the 100 °C experiments (Supplementary Fig. 21) and is in agreement with the previous studies of Harder^7^, and Decarreau et al.^8^. Increasing Mg concentrations in the Fe^3+^ solutions resulted in increased crystallinity of the synthesized products. These results indicate that Fe-rich clay minerals can be precipitated under oxidized conditions, as long as Mg is present in at least small concentrations in the intial starting solution. The pure Mg precipitate had a much broader basal reflection, suggesting less coherent stacking along the c-axis

X-ray diffraction (XRD) and synchrotron micro XRD (μXRD) analysis of synthesized Fe/Mg materials indicate broad low angle 001 peaks, which are characteristic of the large interlayer spacing of smectites (Fig. 1, and Supplementary Figs. 11–13, 24–25, 28–30, 33–35, 37, 39), particularly given the lack of other well-defined peaks that might indicate mica or a peak at a 7 Å spacing which could indicate 1:1 clay minerals^13^. Upon glycolation, synthesized products showed increasing expansion as the Fe^3+^:Mg ratio decreased from 19:1, with the 1:1 ratio material expanding to 17 Å, indicative of a smectite. The range of swelling properties of the precipitates is a characteristic of synthetic high-charge nontronites^8,9,14^ and Fe/Mg smectites (Supplementary Note 1).

XRD analyses indicate variable crystallinity, with the material containing a Fe:Mg ratio of 1:1 having the highest degree of crystallinity based on the basal reflection (Fig. 1). The broad peaks in powder XRD and μXRD indicate that the precipitates formed in this study remained less crystalline than some well-crystalline Fe/Mg-rich clay mineral standards. However, clay minerals in natural terrestrial environments are often less crystalline than reference clay mineral standards^15^, and in some cases exhibit lower crystallinity than the precipitates in this study^16^.

XRD analyses indicate that the b-dimension for these samples was ~ 1.54 Å. This is comparable to the previous work of Russell and Clark^17^, documenting 060 spacings of 1.512 to 1.535 Å for natural nontronites, and to previous measurements of synthetic nontronites that have 060 spacings of ~ 1.54 Å^8,9^. These values have been shown to result from the presence of Fe in both the octahedral and tetrahedral sites, which increases the b-dimension of the clay minerals^10,17^.

μXRD analyses also suggest the presence of ferrihydrite and potentially brucite in the synthesized materials (Supplementary Fig. 42). Although there is some overlap in the diffractions between ferrihydrite and brucite, the increased intensities of the putative brucite in Mg-rich experiments is consistent with the presence of brucite (Supplementary Fig. 42).

### Synchrotron Mössbauer spectroscopy

The ferric state of the Fe-containing precipitates was confirmed by Synchrotron Mössbauer spectroscopy (SMS) (Supplementary Figs. 3–8, Supplementary Table 3). The results of the SMS indicate no evidence of Fe^2+^ within the precipitates, as only one quantum bump/beat is present as well as relatively low isomer shifts, suggesting both sites’ isomer shifts and quadrupole splittings occur within the single observed absorption, which is consistent with the ferric-clay mineral nontronite (Supplementary Figs. 4–8)^18–20^. The quadrupole splitting of all samples are well below that of Fe^2+^-bearing clay minerals^20^, indicating that the samples do not contain measurable Fe^2+^. The Mössbauer parameters determined within the software CONUSS^21^ (Supplementary Table 3) for all samples formed under oxidized conditions are also consistent with the ferric mineral nontronite (standard NAu-1), which was also analyzed and used as a reference in this investigation. The 100-Fe control (subsequently oxidized) had slightly larger isomer shifts and quadrupole splitting than the other experiments (Supplementary Table 3 and Supplementary Fig. 8), but the quadrupole splitting is still below that of Fe^2+^-bearing clay minerals^20^. In addition, our Mössbauer analysis of the 100-Fe control exactly replicated the Mössbauer analysis by Mizutani et al.^9^ of their clay mineral precipitates, which were also shown to be dominated by Fe^3+^. The difference between the 100-Fe control and the other samples may result from the fact that the 100-Fe control initially contained Fe^2+^ rather than Fe^3+^, with resulting differences in Fe-coordination.

### Visible near-infrared and infrared spectroscopy

**Fig. 2:**
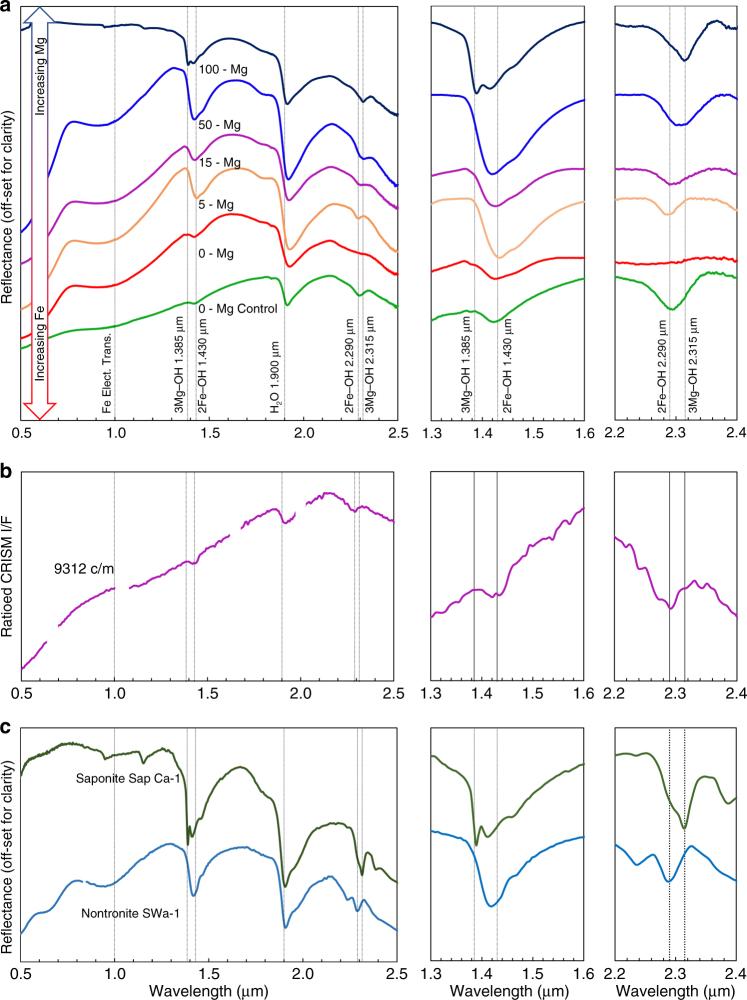
VNIR reflectance spectra of the synthetic clay minerals. **a** VNIR spectra of Fe/Mg-rich clay minerals produced in this study, with Mg concentrations indicated on the figure in percent cation. The 0-Mg control (green) indicates the synthetic nontronite control formed from aqueous ferrous solutions using previously published methods from Mizutani et al.^9^ and Decarreau et al.^8^, and 0-Mg (red) indicates the 100% Fe^3+^ product formed under oxidized conditions. The absorption band for each synthesized material between 1.3–1.6 μm and 2.2–2.4 μm is enlarged for clarity, and has had the continuum removed. Samples with increasing concentrations of Mg (from 0 to 100%) show shifts in the position of hydroxyl-related absorptions as octahedral Fe (2Fe–OH bands at 1.43 μm and 2.285 μm) is replaced by Mg (3Mg–OH bands at 1.385 μm and 2.315 μm)^14,23,24^. **b** VNIR CRISM spectra of Fe-rich smectite from the Nili Fossae region, Mars from Ehlmann et al.^23^ and **c** VNIR spectra of saponite (Mg-rich smectite) and nontronite (Fe^3+^-rich smectite) from the United States Geological Survey spectral library^22^ have similar absorption positions and shapes as the clay minerals precipitated in this study

Syntheses using pure Fe^3+^ solutions with no Mg resulted in an amorphous precipitate (Fig. 1, Supplementary Figs. 20 and 21) with visible near-infrared (VNIR) and infrared (IR) spectra distinctly different from Fe/Mg clay minerals (Fig. 2). Our synthesized Fe/Mg clay minerals, when compared by VNIR and IR at orbital instrument spectral resolution to other synthetic materials, terrestrial analogs, and martian Fe/Mg smectites^22^, are very similar (Fig. 2). With increasing Mg concentration, a band shift from the 2Fe–OH band (2.285 μm) towards the 3Mg–OH bending and stretching vibrations at 2.315 μm occurs^14,23,24^. The structural OH overtone located at ~ 1.410 μm and the fundamental OH-stretch between 2.760 and 2.800 μm also display a systematic shift (Supplementary Figs. 14–17)^14,24^.

The synthetic clay minerals appear red/brown in color (Supplementary Figs. 9, 10, 18, 19, 22, 23, 26, 27, 31, 32, 36 and 38) possibly due to tetrahedrally-coordinated Fe^3+^ (common in terrestrial nontronites^17,25^), but more likely resulting from the presence of small amounts of Fe-oxyhydroxides observed in the µXRD and inductively coupled plasma—optical emission spectroscopy (ICP-OES) results^25,26^, which are also common in terrestrial nontronites^27^. We note that the 50 Mg experiment (the most crystalline of the mixed cation precipitates) produced an absorption at ~650 nm, which contributes to the green color in nontronite.

The presence of charge transfer absorptions and the position of electronic transitions are consistent with ferric clay minerals rather than ferrous for all precipitates in this study (Supplementary Figs. 14 and 16). Broad vibrational absorptions in the VNIR (Supplementary Figs. 14–17), indicate that the precipitates formed in this study remained less crystalline than some well-crystalline Fe/Mg-rich clay mineral standards.

### SEM and EDS analyses

The scanning electron microscopy and energy dispersive spectrosocpy (SEM/EDS) analysis of the precipitates showed that all precipitates were extremely fine-grained, and most of the crystallites were below the resolution of the SEM, making characterization of the morphology difficult (Supplementary Figs. 40 and 41). This observation is in agreement with the XRD data, which indicated a poorly crystalline/fine grained product (also characteristic of terrestrial clay minerals). No other secondary phases were detected with SEM and EDS. However, the elevated concentrations of Fe relative to Si (as determined by EDS) within the samples are consistent with nano phase Fe-oxyhydroxides within the precipitates. If nano phase Fe-oxyhydroxides are present within the sample, they would almost certainly be below the resolution of the SEM/EDS analysis.

### EMP and ICP-OES analyses

The chemical composition of the precipitates was analyzed by electron microprobe (EMP) (Supplementary Table 4), and ICP-OES (Supplementary Table 5). EMP and ICP-OES analyses indicate Fe and Mg enrichment relative to silica (Supplementary Tables 4 and 6). ICP-OES analyses were used to calculate proposed mineral formulae and secondary phases as described in the methods and reported in Supplementary Table 6. Most of the conducted experiments (e.g., the 100-Fe control, 100-Fe^3+^, 5-Mg 95-Fe^3+^, and 15-Mg 85-Fe^3+^ precipitates) have Fe:Mg ratios calculated for the octahedral layer that are >1:1, and because the Fe is in the ferric state with the majority of the octahedral layer filled with trivalent cations, the precipitates are classified as dioctahedral. The remaining experiments (e.g., 50-Mg 50-Fe^3+^, and 100-Mg) have Fe:Mg ratios calculated for the octahedral layer that are approximately equal to or less than 1:1. The 100-Mg experiment has an octahedral layer composed solely of divalent cations and is therefore trioctahedral. Although the 50-Mg 50-Fe^3+^ experiment has a Fe:Mg ratio of the bulk clay mineral of ~1:1, the composition of the octahedral layer calculated as described in the methods does have more Mg than Fe (Supplementary Table 6), and therefore may be more similar to a trioctahedral smectite than a dioctahedral smectite. Because all experiments showed some degree of expansion, the di-octahedral precipitates are referred to as nontronite and the tri-octahedral precipitates as saponite/stevensite.

## Discussion

In this study, synthesis experiments conducted under oxidized conditions in which a divalent cation was present in a concentration of at least 5% led to the precipitation of clay minerals, whereas solely trivalent cations in solution led to a relatively amorphous product. Previous work by Harder^7,11^ has indicated that the divalent cation Fe^2+^ was required for the precipitation of Fe-rich clay minerals, and more recent work by Baldermann et al.^10^ expanded upon this research, also indicating that Fe^2+^ was necessary for the bi-dimensional growth of Fe-bearing clay minerals. In contrast to these previous results, however, our results show that while a divalent cation is necessary, that divalent cation can be Mg^2+^ rather than Fe^2+^. The divalent Mg cation is likely required to stabilize the octahedral layers and promote bi-dimensional growth and thus establish the phyllosilicate structure, playing a similar role to that Fe^2+^ would play if it were present. Magnesium concentrations comprising ~ 5 molar % or greater of the total Fe + Mg component are common in naturally occurring nontronites (Supplementary Table 7). Our results therefore indicate that Fe-clay mineral precipitation can occur under oxidized conditions in the absence of ferrous Fe.

The ranges in chemical composition used in our experiments and common in natural environments^17,28,29^ can cause gradients in crystallinity. XRD analyses of our precipitates indicate variable crystallinity, with the material containing a Fe:Mg ratio of 1:1 having the highest degree of crystallinity based on the basal reflection (Fig. 1). Upon glycolation, synthesized products similarly showed increasing expansion as the Fe^3+^:Mg ratio decreased from the most Fe-rich precipitates, with the 1:1 ratio material expanding to 17 Å, indicative of classic smectite behavior. Previous work by Grauby et al.^14^ synthesizing the nontronite-saponite series at higher temperatures over a longer time period does not report a significant gradient in crystallinity along the Fe^3+^–Mg compositional gradient comparable to that which we see in our experiments. However, although all precipitates from Grauby et al.^14^ were crystalline, based on our examination of their published data, the most Fe-rich and Mg-rich samples were less crystalline, where the Fe-rich sample had decreased crystallinity indicated in the *0k0* reflection, and the Mg-rich sample had decreased crystallinity along the *00l* (e.g., the *c*-axis). Intermediate Fe:Mg ratio experiments, in contrast, were more crystalline. Therefore, our examination of their results suggests a similar trend in crystallinity with changes in composition to that observed in our experiments.

The VNIR spectra of our precipitates (Fig. 2a) indicate that materials with varying degrees of crystallinity such as those precipitated in our experiments and common in natural terrestrial soils^15,16^ may produce spectra similar to well-crystalline clay minerals with comparable chemical compositions (Fig. 2c). Our synthesized Fe/Mg clay minerals, when compared by VNIR and IR at orbital instrument spectral resolution to martian Fe/Mg smectites^22^, are also very similar (Fig. 2b). Milliken et al.^30^ have also shown that the poorly/nano crystalline material hisingerite has VNIR spectral properties similar to nontronite. X-ray amorphous or poorly/nano crystalline phases are a significant component of the samples analyzed by MSL at Gale Crater^31^, and our results indicate that materials on Mars currently identified as clay minerals from orbit may also include poorly/nano crystalline materials.

Our results can also help interpret the chemical composition of Fe/Mg-rich materials from orbit. Increasing concentrations of Mg (from 0 to 100%) show a band shift from the 2Fe–OH band (2.285 μm) towards the 3Mg–OH bending and stretching vibrations at 2.315 μm^14,23,24^. A systematic shift is also evident in the structural OH overtone located at ~ 1.410 μm and the fundamental OH-stretch between 2.760 and 2.800 μm (Supplementary Note 1)^14,24^. In combination, these absorption features can be used to interpret the ratio of Fe to Mg in clay minerals observed from orbit on Mars (Fig. 2).

Synthesis experiments can also help us better interpret past aqueous conditions on Mars, including the chemical composition, the oxidation state, and the duration and temperature of alteration. Increasing the duration of ageing in experiments (corresponding to the time of water–rock interaction) increases the crystallinity of the precipitates^10,32^. Warmer temperatures over the same duration also increase crystallinity^8^. Thus, either shorter durations of water–rock interaction and/or colder temperatures may be implicated if Fe-rich clay minerals on Mars are, like some of our synthesized materials, poorly crystalline. In addition, the presence of more poorly crystalline materials is also consistent with a lack of significant water–rock interaction after their precipitation^33^.

Seeking possible signs of life, including organic biosignatures, is a crucial part of the Mars Exploration Program. Our work demonstrated that dioctahedral Fe-rich clay minerals, such as nontronite, of variable crystallinity can form rapidly under oxidized conditions in the presence of at least small amounts of Mg. Oxidized conditions would not favor the preservation of organic matter in the primary environment of clay mineral precipitation. Since Fe-rich smectites containing Mg are widespread on Mars^6,34–37^, these results suggest that clay mineral-bearing terrains may not always be conducive to the preservation of organic matter, and could help explain low concentrations of organic compounds in some smectite-bearing rocks explored by the Curiosity rover at Gale Crater^38,39^.

## Methods

### Mineral synthesis

In order to better understand clay mineral-forming environments on Mars and the potential for the preservation of organic matter, we performed synthesis experiments of clay minerals with a range of chemical compositions. Experiments were performed under oxidized and basic conditions (Table 1). Initial silica-containing solutions were made acidic to dissolve Fe^3+^- and Mg-sulfates, and were then made basic by the addition of NaOH. This process is likely analogous to the interaction of acidic, ferric sulfate-containing solutions, such as those proposed on Mars^40^, with silica-containing minerals, resulting in an increased solution pH. The solutions were then incubated at two different temperatures to test the effects of temperature. In addition, a synthetic nontronite control was synthesized using standard methods from Fe^2+^-containing solutions^8,9^. Detailed experimental methods are provided immediately below.

Following a method similar to that of Mizutani et al.^9^, 4.35 g of sodium meta-silicate was dissolved in 420 mL of 18.2 MΩ water. The solution was then acidified with 0.5 M H_2_SO_4_ to a pH of ~3. For the synthetic nontronite control only, 4.2 g of sodium dithionite was dissolved in the solution to maintain reducing conditions. Various concentrations (Table 1 and Supplementary Tables 1 and 2) of reagent-grade Fe and Mg sulfates (mineralogy identified by XRD shown in Supplementary Figs. 1 and 2) were then dissolved in the acidic solution. The solution was cleared (i.e., a precipitate formed and the solution left clear) by the addition of 19.8 mL of 5 M NaOH. The prepared suspensions were aged under ambient conditions for 1 day at ~20 °C, then subsequently heated at either 150 °C for 48 h in Teflon lined Parr-vessels (Supplementary Table 1), or 100 °C in high-density polyethylene bottles for 60 days (Supplementary Table 2). At the end of each experiment, the pH of the solution was measured with an S20 SevenEasy pH meter using a two-point calibration. Samples were then vacuum-filtered to obtain the solid particulate using a Pyrex 47 mm, Microfiltration all-glass assembly with a 300 mL funnel and 47 mm, 0.45 μm membrane disc filters (as recommended by Moore and Reynolds^16^ and following the methodology of Mizutani et al.^9^). Samples were then dried in-vacuo in a desiccator. Filtration might favor the collection of larger crystallites/particulates, as any crystallite/particle smaller than 0.45 µm may pass through the filter, which might increase the observed overall crystallinity of the precipitates.

### Redox condition of experiments

As shown in Supplementary Table 1, we added ferric sulfates to the experiments, and ferrous sulfates to the nontronite control. The oxidation state of the reagents was confirmed by measuring them by XRD (Supplementary Figs. 1 and 2), and the oxidation state of the iron within our synthesized precipitates using SMS. We measured four samples using SMS: the clay mineral standard NAu-1 and experiments 100-Fe Control, 15-Mg 85-Fe, and 50-Mg 50-Fe—further analytical details are given below. All SMS analyses of the precipitates showed the sample to be 100% ferric, with any ferrous Fe present below the detection limit of SMS (see SMS results section and Supplementary Figs. 3–8 and Supplementary Table 3). The minimum oxidation state of the solutions based on Fe concentrations and an extremely conservative upper limit of the potential Fe^2+^ contamination in our initial ferric starting materials of 3% is 0.856 V, and ferrous Fe is extremely rapidly oxidized under these high pH, oxidized conditions^42,43^.

### Characterization and analytical techniques

Synthesized materials were analyzed with powder XRD, synchrotron μXRD, SMS, VNIR Spectroscopy, SEM with EDS, EMP analysis, and ICP-OES.

### Powder X-ray diffraction

The powder XRD data were collected for the synthesized samples using a PANalytical X′Pert Pro X-Ray Diffractometer at 40 kV and 20 mA using CuKα radiation in the X-Ray Fluorescence and X-Ray Diffraction Laboratory (XXL) at the University of Nevada, Las Vegas. The analyzed samples were prepared in a similar manner to the glass slide method illustrated by Moore and Reynolds^16^ in which samples were lightly ground in the presence of ethanol. The slurry was then poured onto a silicon wafer and dried at 60 °C. Patterns were taken under air dried conditions, as well as after treatment with ethylene glycol vapor for a minimum of 24 h to measure expansion and therefore identify the specific clay mineralogy.

Clay minerals are generally fine-grained, crystalline, hydrous phyllosilicates^16^. Of these minerals, the smectite and the vermiculite group are defined by their ability to expand and shrink when exposed to heat, water and polar organic compounds (vermiculite to a lesser extent than smectites)^16^. If expansion was observed, a trait reserved for smectites and vermiculites, the clay mineral was described as a high-charge smectite to encompass both smectite and vermiculite properties, and if the clay mineral expanded to ≥ 17 Å that clay mineral was described as a smectite.

### Synchrotron micro X-ray diffraction

Select aliquots of powdered sample were placed in 1 mm capillary tubes and examined with μXRD. The μXRD was conducted at the 16-ID-D beamline of the Advanced Photon Source in the Argonne National Laboratory using monochromatic radiation with a wavelength of 0.860250 Å and also at beamline 12.2.2 of the Advanced Light Source (ALS). The beam was focused to a 30 × 40 μm^2^ spot at the sample position. A MAR165 area detector was used for collecting the diffraction data. Sample detector distance and geometric distortions were determined based on a CeO_2_ standard using GSE-ADA^43^. The diffraction patterns were integrated using Fit2D^44^.

### Synchrotron Mössbauer spectroscopy

In order to determine the oxidation state of the Fe within the precipitates, SMS was performed on selected samples (NAu-1, 100-Fe control, 15-Mg 85-Fe^3+^, and 50-Mg 50-Fe^3+^) at the 16-ID-D beamline of the Advanced Photon Source at Argonne National Laboratory using monochromatic radiation of 14.4 kV. Collection times were a minimum of 1 h, using a 10 μm steel foil to determine center shifts (Supplementary Fig. 3). In order to determine Mössbauer parameters, fitting was performed using the software CONUSS^21^ (Supplementary Figs. 4–8). As with previous Mössbauer studies of nontronite, spectra were modeled with two Mössbauer sites^18,20^. The octahedral sites of clay minerals contain four oxygen ligands and the remaining two are hydroxyls, which may be in either *cis* or *trans* configuration^20^ and therefore require two Mössbauer sites.

### Visible near-infrared and infrared spectroscopy

VNIR and IR reflectance spectra were measured for each sample, using an Analytical Spectra Devices (ASD) VNIR spectrometer and a Fourier Transform Infrared Spectrometer, respectively. Spectra were taken over the range of 0.4–2.5 μm and 2.5–25 μm. The spectra of our samples are similar to spectra measured by Observatoire pour la Mineralogie, l’Eau les Glaces et l’Activité (OMEGA), the Compact Reconnaissance Imaging Spectrometer for Mars (CRISM), the Thermal Emission Spectrometer (TES), and Mini-TES, and are therefore comparable to remote and ground-based observation of the martian surface^42^. VNIR band centers were determined from continuum-removed spectra.

### SEM and EDS analyses

SEM was performed using a JEOL JSM-5610 scanning electron microscope equipped with an Oxford ISIS Energy Dispersive Spectrometer (EDS) capable of semi-quantitative chemical analysis. Additional SEM analyses were performed using a LEO 1550 SFEG scanning electron microscope, equipped with an energy dispersive X-ray spectrometer. Samples were carbon-coated and examined for morphology and chemical composition. SEM/EDS analyses were conducted at the University of Nevada, Las Vegas Electron Microanalysis and Imaging Laboratory (EMiL) and at Stony Brook University’s Materials Characterization Laboratory.

### Microprobe

Analysis by EMP-WDS was carried out on a Jeol JXA-8900 microprobe at the University of Nevada, Las Vegas EMiL laboratory on polished epoxy mounts. Analysis conditions were 20 keV and 10 nA using a 10 μm beam.

The difficulties encountered when analyzing clay minerals by EMP analysis and the low totals observed (Supplementary Table 4) have been previously documented^46^. Clay minerals may undergo dehydration under desiccation (during the carbon coating process and within the vacuum chamber of the microprobe itself) but retain structural OH, which may contain ~ 5 weight percent H_2_O^46^. In addition, EMP analysis relies on a relatively flat sample surface; this can be difficult to achieve due to etching and pitting during the polishing process, which was also observed by Treiman et al.^46^. Clay minerals are also highly porous, which may contribute to low totals observed by EMP analysis^46^ (Supplementary Table 4).

### ICP-OES analyses

The chemical composition of the precipitates was determined by ICP-OES after acid digestion. Acid digestion of the samples was achieved using previously published methods^47^ described as follows. Samples were mixed with lithium borate (1:5 ratio), and heated in an oven at 975 °C for 10 min, shaken (to ensure proper mixing), and then heated for an additional 10 min. Upon removal from the oven, the mixture was poured directly into 100 mL of 1 M nitric acid, and stirred for 1 h. The solution was then diluted with additional 1 M nitric acid to the desired concentration, below the point of silica polymerization, and then samples were stirred overnight to ensure homogeneity and total dissolution.

ICP-OES analyses were performed at Stony Brook University. Elemental concentrations (Al, Ca, Fe, Mg, Na, Si, and Ti) within the solutions were determined with an iCAP 6300 radial view Inductively Coupled Plasma—Optical Emission Spectrometer (Supplementary Table 5). Standards were matrix matched with nitric acid for elemental analysis. Proposed chemical formulae for the synthetic Mg–Fe clay minerals and the estimated amounts of the presumed secondary phases ferrihydrite and brucite were calculated for each of the 150 °C experiments from the bulk composition of the precipitates (Supplementary Table 5), and are presented in Supplementary Table 6. In order to generate mineral percentages and mineral formulae, we assume that the interlayer charge is 0.25 (for a O_10_(OH)_2_ half unit cell) for all samples that expanded to the classic ~ 17 Å upon glycolation (100-Mg and the 50-Mg 50-Fe^3+^). An interlayer charge of 0.75 is used for samples that did not expand to ~ 17 Å upon glycolation^8^. Fe and Mg were added at the ratio determined through ICP-OES to the octahedral and tetrahedral sites to balance the required charge. Because Mg does not reside within tetrahedral sites^16^, Mg was given priority when assigning Mg and Fe to the octahedral sites. The remaining Fe not present in the octahedral layer was then placed within the tetrahedral sites. The remaining Fe and Mg not assigned to the clay mineral structure was portioned into the secondary phases observed by μXRD (e.g., ferrihydrite and brucite). The resulting Fe:Mg ratio of the bulk clay minerals is within 1% (molar ratio) of the initial solution chemistry. The lower ratios of Si to Fe and Mg may have resulted from the incomplete solubilization of Si, as HF was not added during the digestion^47^.

The dioctahedral Fe^3+^ end-member of the smectite group is nontronite, whereas the trioctahedral Mg end-member is either saponite or stevensite, where the charge is derived from tetrahedral and octahedral substitution, respectively^16^. However, significant Fe and Mg substitution can occur between these two end-members, leading to a semi-solid solution in Fe/Mg-smectites and other clay minerals^14^. In addition, previous work by Grauby et al.^14^ also suggests that di- and trioctahedral domains may occur within the same crystallite. The di / trioctahedral nature of the precipitates of this study were assigned based on the molar ratio of di- and trivalent cations within the octahedral layer calculated as described above (e.g. if the octahedral layer is dominated by ferric Fe the clay mineral is dioctahedral, whereas if it is dominated by Mg it is trioctahedral).

### Data availability

The authors declare that the data supporting the finding of this study are available within the paper and its supplementary materials, and/or from the corresponding author S.R.G. upon reasonable request.

## Electronic supplementary material

Supplementary Information

## References

[1] Bishop JL (2008). Phyllosilicate diversity and past aqueous activity revealed at Mawrth Vallis, Mars. Science.

[2] Carter J, Poulet F, Bibring JP, Mangold N, Murchie S (2013). Hydrous minerals on Mars as seen by the CRISM and OMEGA imaging spectrometers: Updated global view. J. Geophys. Res. Planets.

[3] Ehlmann BL, Mustard JF, Clark RN, Swayze GA, Murchie SL (2011). Evidence for low-grade metamorphism, hydrothermal alteration, and diagenesis on Mars from phyllosilicate mineral assemblages. Clays Clay Miner..

[4] Ehlmann BL (2008). Clay minerals in delta deposits and organic preservation potential on Mars. Nat. Geosci..

[5] Mustard JF (2008). Hydrated silicate minerals on Mars observed by the Mars Reconnaissance Orbiter CRISM instrument. Nature.

[6] Michalski JR (2015). Constraints on the crystal-chemistry of Fe/Mg-rich smectitic clays on Mars and links to global alteration trends. Earth. Planet Sci. Lett..

[7] Harder H (1976). Nontronite synthesis at low temperatures. Chem. Geol..

[8] Decarreau A (2008). Hydrothermal synthesis, between 75 and 150 C, of high-charge, ferric nontronites. Clays Clay Miner..

[9] Mizutani T, Fukushima Y, Okada A, Kamigaito O, Kobayashi T (1991). Synthesis of 1: 1 and 2: 1 iron phyllosilicates and characterization of their iron state by Mössbauer spectroscopy. Clays Clay Miner..

[10] Baldermann A (2014). The Fe-Mg-saponite solid solution series–a hydrothermal synthesis study. Clay Miner..

[11] Harder H (1978). Synthesis of iron layer silicate minerals under natural conditions. Clays Clay Miner..

[12] Decarreau A, Bonnin D, Badaut-Trauth D, Couty R, Kaiser P (1987). Synthesis and crystallogenesis of ferric smectite by evolution of Si-Fe coprecipitates in oxidizing conditions. Clay Miner..

[13] Vaniman D (2014). Mineralogy of a mudstone at Yellowknife Bay, Gale crater, Mars. Science.

[14] Grauby O, Petit S, Decarreau A, Baronnet A (1994). The nontronite-saponite series: an experimental approach. Eur. J. Mineral..

[15] Hausrath E, Navarre-Sitchler A, Sak P, Williams J, Brantley S (2011). Soil profiles as indicators of mineral weathering rates and organic interactions for a Pennsylvania diabase. Chem. Geol..

[16] Moore, D. M. & Reynolds, R. C. *X-ray Diffraction and the Identification and Analysis of Clay Minerals* Vol. 378 (Oxford university press Oxford, 1989).

[17] Russell J, Clark D (1978). The effect of Fe-for-Si substitution on the b-dimension of nontronite. Clay. Miner..

[18] Ribeiro FR, Fabris JD, Kostka JE, Komadel P, Stucki JW (2009). Comparisons of structural iron reduction in smectites by bacteria and dithionite: II. A variable-temperature Mössbauer spectroscopic study of Garfield nontronite. Pure Appl. Chem..

[19] Taylor GL, Ruotsala A, Keeling R (1968). Analysis of iron in layer silicates by Mössbauer spectroscopy. Clays Clay Miner..

[20] Vandenberghe, R. E. & De Grave, E. in *Application of Mössbauer Spectroscopy in Earth Sciences* 91-185 (Springer Berlin Heidelberg 2013).

[21] Sturhahn W (2000). CONUSS and PHOENIX: Evaluation of nuclear resonant scattering data. Hyperfine. Interact..

[22] Clark, R. N. et al. *USGS Digital Spectral Library Splib06a* (US Geological Survey Reston, VA, 2007).

[23] Ehlmann, B. L. et al. Identification of hydrated silicate minerals on Mars using MRO‐CRISM: Geologic context near Nili Fossae and implications for aqueous alteration. *J. Geophys. Res. Planets* **114** E00D08 (2009).

[24] Bishop J, Murad E, Dyar M (2002). The influence of octahedral and tetrahedral cation substitution on the structure of smectites and serpentines as observed through infrared spectroscopy. Clay Miner..

[25] Merola RB, McGuire MM (2009). Crystallographic site distribution and redox activity of Fe in nontronites determined by optical spectroscopy. Clays Clay Miner..

[26] Brigatti MF (1983). Relationships between composition and structure in Fe-rich smectites. Clay Miner..

[27] Murad E (1987). Mössbauer spectra of nontronites: structural implications and characterization of associated iron oxides. Z. Pflanzenernähr. Bodenkd..

[28] Manceau A (2000). Oxidation-reduction mechanism of iron in dioctahedral smectites: I. Crystal chemistry of oxidized reference nontronites. Am. Mineral..

[29] Goodman B, Russell J, Fraser A, Woodhams F (1976). A Mössbauer and IR spectroscopic study of the structure of nontronite. Clays Clay Miner..

[30] Milliken R, Bish D (2014). Distinguishing Hisingerite from Other Clays and its Importance for Mars. 45th Lun.. Plan. Sci. Con..

[31] Bish DL (2013). X-ray diffraction results from Mars Science Laboratory: Mineralogy of Rocknest at Gale crater. Science.

[32] Baker LL, Strawn DG (2014). Temperature effects on the crystallinity of synthetic nontronite and Implications for nontronite formation in Columbia river basalts. Clays Clay Miner..

[33] Tosca NJ, Knoll AH (2009). Juvenile chemical sediments and the long term persistence of water at the surface of Mars. Earth Planet Sci. Lett..

[34] Bishop JL (2013). What the ancient phyllosilicates at Mawrth Vallis can tell us about possible habitability on early Mars. Planet Space Sci..

[35] Ehlmann BL (2011). Subsurface water and clay mineral formation during the early history of Mars. Nature.

[36] Michalski J, Poulet F, Bibring J-P, Mangold N (2010). Analysis of phyllosilicate deposits in the Nili Fossae region of Mars: Comparison of TES and OMEGA data. Icarus.

[37] Poulet F (2005). Phyllosilicates on Mars and implications for early martian climate. Nature.

[38] Freissinet C (2015). Organic molecules in the sheepbed mudstone, gale crater, mars. J. Geophys. Res.Planets.

[39] Ming D (2014). Volatile and organic compositions of sedimentary rocks in Yellowknife Bay, Gale crater, Mars. Science.

[40] Tosca NJ, McLennan SM, Lindsley DH (2004). Acid‐sulfate weathering of synthetic Martian basalt: The acid fog model revisited. J. Geophys. Res. Planets.

[41] Millero FJ, Sotolongo S, Izaguirre M (1987). The oxidation kinetics of Fe(II) in seawater. Geochimica et Cosmochimica Acta.

[42] Stumm W, Lee GF (1061). Oxygenation of ferrous iron. Ind. Eng. Chem..

[43] Dera P (2013). High pressure single-crystal micro X-ray diffraction analysis with GSE_ADA/RSV software. High Pressure Research.

[44] Hammersley, A. FIT2D: an introduction and overview. *European Synchrotron Radiation Facility Internal Report* Report No. ESRF97HA02T, (1997).

[45] Ehlmann B, Bish D, Ruff S (2012). Volatile and organic compositions of sedimentary rocks in Yellowknife Bay, Gale crater, Mars. J. Geophys. Res. Planets.

[46] Treiman AH (2014). Ferrian saponite from the Santa Monica Mountains (California, USA, Earth): Characterization as an analog for clay minerals on Mars with application to Yellowknife Bay in Gale Crater. Am. Mineral..

[47] Potts P, Webb P, Watson J (1984). Energy‐dispersive x‐ray fluorescence analysis of silicate rocks for major and trace elements. X‐Ray Spectrometry.

