# Supplementary material for: Clay mineral formation under oxidized conditions and implications for paleoenvironments and organic preservation on Mars
Source: Nat Commun. 2017 Nov 1;8:1230. doi: 10.1038/s41467-017-01235-7 (PMC5663933; doi:10.1038/s41467-017-01235-7)
Supplement: Supplementary file 1 — Supplementary Information [file 41467_2017_1235_MOESM1_ESM.pdf]

Supplementary Note 1

Experiments performed at 100º C and 150º C were very similar, and are therefore discussed together below.

0-Mg 100-Fe^{3+} Control - Subsequently Oxidized

The 100-Fe control directly replicated the previous work of Mizutani et al.[1], in which nontronite was synthesized under initially reducing conditions and subsequently oxidized through exposure to atmospheric conditions with oxidation confirmed by Mössbauer spectroscopy[1,2]. Trioctahedral ferrous smectites are very unstable under oxidizing conditions and have been shown to oxidize during desiccation under laboratory conditions[3]. The initial 150º C precipitate was dark green in color and slowly became reddish brown in color, which may be an indicator of the oxidation of Fe from ferrous to ferric (Figure S9). In contrast, the 100º C experiment appeared to be slightly greener in color, but still was a reddish orange color upon drying and crushing (Figure S10). Diffraction patterns of the synthesized products included a broad low angle peak of approximately 12.5 Å, indicative of a smectite clay mineral (Figure S11 (150º C) and Figure S12 (100º C)). The lack of other peaks at or near 7 Å suggests the absence of other clay minerals such as kaolins and chlorites. The µXRD analyses of the 150º C synthesized material also showed distinct reflections characteristic of clay minerals (e.g. 001, 020 and 060) (Figure S13 (150º C)). Treatment with ethylene glycol produced only a slight expansion (~ 13-14 Ǻ) more characteristic of a high-charge smectite / vermiculite, which expands very little when exposed to ethylene glycol vapor (Figure S11 (150º C) and Figure 12 (100º C)[2]. A VNIR absorption feature at 0.975 µm is due to Fe electronic transitions which have a distinctive overall continuum slope toward shorter wavelengths, distinct from the other samples (Figure S14 and S15 (150º C) and S16 and S17 (100º C)). The absorption feature at 1.422 µm is

characteristic of OH and H_2O (2vOH) features of metal-hydroxyl bonds and structural and absorbed water. In addition, an absorption feature located at 1.914 μm is indicative of H_2O (VOH + δm-OH). The M-OH (2vOH) absorption feature was located at 2.294 μm, a somewhat longer wavelength than expected for the Fe(III)-smectite nontronite (2.28-2.29 μm).

0-Mg 100-Fe^{3+}

The initial precipitate was dark red in color (Figure S18 (150º C) and S19 (100º C)). Diffraction patterns of the synthesized products suggest the precipitates remained relatively amorphous (Figure S20 (150º C) and S21 (100º C)), which is supported by previous studies suggesting that Fe-dioctahedral clay mineral formation from ferric solutions only is prohibitively slow and difficult[2,4] due to the absence of a divalent cation to stabilize the octahedral layers, a necessity for the early bi-dimensional growth of these clay minerals[4-6]. Treatment with ethylene glycol had little if any effect (Figure S20 (150º C)). An absorption feature at 1.013 μm indicates Fe electronic transitions (Figure S14 and S15 (150º C) and S16 and S17 (100º C)). The absorption feature at 1.426 μm is characteristic of OH and H_2O (2vOH) features of metal-hydroxyl structural and absorbed water. This absorption feature is close to that of nontronite (1.43 μm). In addition, an absorption feature located at 1.925 μm is indicative of H_2O (VOH + δm-OH). This absorption feature is broad and shallow, which may suggest a lack of Fe-OH structural elements and/or poorly crystalline components.

100-Mg 0-Fe^{3+}

The initial precipitate was white in color (Figure S22 (150º C) and S23 (100º C)). Diffraction patterns of the synthesized products contained broad low angle peaks (Figure S24 (150º C) and S25 (100º C)) that made it difficult to determine an accurate *001* d-spacing of the

150º C experiment (Figure S24). After treatment with ethylene glycol, expansion occurred, although the exact expansion is difficult to determine in the 150º C experiment (Figure S24); the 100º C experiment expanded from approximately 12 to 19 Å (Figure S25). These data are consistent with a Mg-trioctahedral smectite (e.g. saponite or stevensite). The VNIR spectra of the experiments contain several vibrational absorptions located at 1.157 and 1.281 μm that are likely due to structural H_2O (Figure S14 and S15 (150º C), and S16 and S17 (100º C)). The absorption feature at 1.389 and 1.417 μm is characteristic of Mg-OH and H_2O (2vOH) features of structural and absorbed water. An absorption feature located at 1.914 μm is indicative of H_2O (VOH + δm-OH). The M-OH (2vOH) absorption feature is located at 2.314 μm, indicating Mg-OH vibrations. The spectral properties are also consistent with a Mg-trioctahedral smectite (e.g. saponite or stevensite).

15-Mg 85-Fe^{3+}

The initial precipitate was red-brown in color (Figure S26 (150º C) and S27 (100º C)). The diffraction pattern of the synthesized material contained a broad low-angle peak, indicative of a clay mineral and also showed other distinct reflections of clay minerals (e.g. 020 reflection) (Figure S28 (150º C) and S29 (100º C)). These results were also supported by μXRD for the 150° C experiment (Figure S30). In addition, treatment with ethylene glycol resulted in some expansion, which is a property of smectites, although due to the poor/nano crystallinity the exact expansion is difficult to determine (Figure S28 (150º C) and Figure S29 (100º C)). These data are indicative of a Fe-rich dioctahedral clay mineral, such as a high-charge nontronite[2]. In the VNIR spectra, an absorption feature at 1.009 μm indicates Fe electronic transitions (Figure S14 and 15 (150º C), and S16 and S17 (100º C)). The absorption feature at 1.424 μm is characteristic of OH and H_2O (2vOH) features of structural and absorbed water, and this absorption feature is

suggestive of nontronite when at 1.43 μm, although band shifts may occur as a result of multiple cations within the mineral structure. An absorption feature located at 1.921 μm is indicative of H_2O (VOH + δm-OH). The M-OH (2vOH) absorption feature is located at 2.295 μm. This absorption feature is closest to that of nontronite (2.28 - 2.29 μm). The VNIR absorptions of the 15-Mg 85-Fe^{3+} experiments are slightly broader than some terrestrial samples, however the "L-shape" with a shoulder rather than a sharp absorption is a common characteristic of Fe/Mg phyllosilicate-bearing terrain observed with CRISM and OMEGA (e.g. Poulet et al.[7]).

50-Mg 50-Fe^{3+}

The initial precipitate was red-brown in color (Figure S31 (150º C) and S32 (100º C)). Diffraction patterns of the synthesized products contained broad, low angle peaks at approximately 14 -15 Å, indicative of a smectite clay mineral (Figure S33 (150º C) and Figure S34 (100º C)). These results were also supported by μXRD for the 150º C experiment indicating the presence of 020 and 060 reflections of clay minerals (Figure S35). Treatment with ethylene glycol produced an expansion to ~17 Å indicative of smectite (Figure S33 (150º C)). However, the 100º C precipitate only expanded to approximately 15 Å (Figure S34 (100º C)). These precipitates may contain domains of both Mg-trioctahedral and Fe-dioctahedral clay minerals/smectites[8]. An absorption feature at 0.984 μm is due to Fe electronic transitions (~1 μm) (Figure S14 and S15 (150º C), and S16 and S17 (100º C)). The absorption feature at 1.420 μm is characteristic of OH and H_2O (2vOH) features of hydroxyl and structural and absorbed water. This absorption feature's center wavelength is between that of montmorillonite and nontronite (1.41 and 1.43 μm). In addition, an absorption feature located at 1.920 μm is indicative of H_2O (VOH + δm-OH). The M-OH (2vOH) absorption feature is located at 2.309 μm, but is broad and ranges from 2.300 to 2.318 μm. This absorption feature occurs between

that of nontronite (2.28 – 2.29 μm) and saponite (2.31 – 2.32 μm), although it is closer to that of saponite. The VNIR absorptions of 50-Mg 50-Fe^{3+} experiments are slightly broader than well-crystalline terrestrial samples (Figures S14 and S16), which is also a common characteristic of Fe/Mg phyllosilicate-bearing terrain observed with CRISM and OMEGA (e.g. Poulet et al.[7]).

10-Mg 90-Fe^{3+}

The initial precipitate was red-brown in color (Figure S36). Diffraction patterns of the synthesized product contained a broad, low angle peak, indicative of clay minerals (Figure S37). The diffraction pattern also contained a 020 reflection indicative of clay minerals. Treatment with ethylene glycol resulted in only a slight expansion of the basal plane, roughly expanding from 13 Å to approximately 14 Å. The diffraction data are similar to materials previously described as high-charge nontronites[2]. A VNIR absorption feature at 0.975 μm is due to Fe electronic transitions (Figure S14 and S15). The absorption feature at 1.433 μm is characteristic of OH and H_2O (2vOH) features of metal-hydroxyl bonds and structural and absorbed water and coincides with the absorption of nontronite (1.43 μm). In addition, an absorption feature located at 1.914 μm is indicative of H_2O (VOH + δm-OH). The M-OH (2vOH) absorption feature was located at 2.289 μm, indicative of the clay mineral nontronite (2.28 – 2.29 μm). The VNIR absorptions of the 10-Mg 90-Fe^{3+} material are slightly broader than well-crystalline terrestrial samples (Figures S14 and S16), which is also a common characteristic of Fe/Mg phyllosilicate-bearing terrain observed with CRISM and OMEGA (e.g. Poulet et al.[7]).

5-Mg 95-Fe^{3+}

The initial precipitate was red-brown in color (Figure S38). Diffraction patterns of the synthesized product contained a broad, low angle peak, indicative of a clay mineral (Figure S39).

Although it was difficult to determine the exact position of the basal plane due to poor/nano crystallinity, when treated with ethylene glycol expansion appears to have occurred (Figure S39). The diffraction pattern also contained the 020 reflection indicative of clay minerals. Only 5% Mg was used in the synthesis of this precipitate, which therefore indicates that only very minor amounts of Mg are required in the formation of Fe-rich clay minerals such as this high-charge nontronite[2]. Many naturally occurring nontronites contain a similar amount of Mg[9-12] (Table S5). A VNIR absorption feature at 0.975 μm is due to Fe electronic transitions (Figure S14 and S15). The absorption feature at 1.434 μm is characteristic of OH and H_2O (2vOH) features of metal-hydroxyl bonds and structural and absorbed water and coincides with the absorption in nontronite (1.43 μm). In addition, an absorption feature located at 1.914 μm is indicative of H_2O (VOH + δm-OH). The M-OH (2vOH) absorption feature was located at 2.287, indicative of the clay mineral nontronite (2.28 – 2.29 μm). The VNIR absorptions of the 5-Mg 95-Fe^{3+} experiments are slightly broader than well crystalline terrestrial samples, which is also a common characteristic of Fe/Mg phyllosilicate-bearing terrain observed with CRISM and OMEGA (e.g. Poulet et al.[7]).

Supplementary Table 1: Concentration of reagent grade chemicals that were incorporated into solution to produce the synthesized products at 150° C

100-Fe Control

Chemical Name	Molecular Weight (g/mol)	Amount (g or mL)	Amount Cation (mol)
Sodium Metasilicate - Pentahydrate	212.7400	4.35	0.0204
Iron (II) Sulfate - Heptahydrate	278.0500	3.94	0.0142
Sodium Dithionite	174.1070	4.20	0.0241
**Fe:Mg ratio**	-	-	1:0

Solution Name	Molecular Weight (g/mol)	Amount (mL)	Amount Cation (mol)
Sodium Hydroxide (5 M)	-	19.80	-
H_2O	18.0153	417.52	-
Sulfuric Acid (0.5 M)	-	41.00	-
**Ending pH**	-	-	12.49

100-Fe^{3+}

Chemical Name	Molecular Weight (g/mol)	Amount (g or mL)	Amount (mol)
Sodium Metasilicate - Pentahydrate	212.7400	4.35	0.0204
Iron (III) Sulfate - Rhomboclase & Ferricopiapite	321.0340	4.02	0.0138
**Fe:Mg ratio**	-	-	1:0

Solution Name	Molecular Weight (g/mol)	Amount (mL)	Amount Cation (mol)
Sodium Hydroxide (5 M)	-	19.80	-
H_2O	18.0153	419.96	-
Sulfuric Acid (0.5 M)	-	42.00	-
**Ending pH**	-	-	12.69

100-Mg

Chemical Name	Molecular Weight (g/mol)	Amount (g or mL)	Amount (mol)
Sodium Metasilicate - Pentahydrate	212.7400	4.36	0.0205
Magnesium Sulfate - Epsomite	246.4700	3.49	0.0142
**Fe:Mg ratio**	-	-	0:1

Solution Name	Molecular Weight (g/mol)	Amount (mL)	Amount Cation (mol)
Sodium Hydroxide (5 M)	-	19.80	-
H_2O	18.0153	417.87	-
Sulfuric Acid (0.5 M)	-	42.00	-
**Ending pH**	-	-	12.88

15-Mg 85-Fe^{3+}

Chemical Name	Molecular Weight (g/mol)	Amount (g or mL)	Amount (mol)
Sodium Metasilicate - Pentahydrate	212.7400	4.36	0.0205
Iron (III) Sulfate - Rhomboclase & Ferricopiapite	321.0340	3.42	0.0117
Magnesium Sulfate - Epsomite	246.4700	0.53	0.0022
**Fe:Mg ratio**	-	-	17:3

Solution Name	Molecular Weight (g/mol)	Amount (mL)	Amount Cation (mol)
Sodium Hydroxide (5 M)	-	19.80	-
H_2O	18.0153	416.41	-
Sulfuric Acid (0.5 M)	-	42.00	-
**Ending pH**	-	-	12.76

50-Mg 50-Fe^{3+}

Chemical Name	Molecular Weight (g/mol)	Amount (g or mL)	Amount (mol)
Sodium Metasilicate - Pentahydrate	212.7400	4.35	0.0204
Iron (III) Sulfate - Rhomboclase & Ferricopiapite	321.0340	2.02	0.0069
Magnesium Sulfate - Epsomite	246.4700	1.76	0.0071
**Fe:Mg ratio**	-	-	1:1

Solution Name	Molecular Weight (g/mol)	Amount (mL)	Amount Cation (mol)
Sodium Hydroxide (5 M)	-	19.80	-
H_2O	18.0153	417.11	-
Sulfuric Acid (0.5 M)	-	42.00	-
**Ending pH**	-	-	12.79

10-Mg 90-Fe^{3+}

Chemical Name	Molecular Weight (g/mol)	Amount (g or mL)	Amount (mol)
Sodium Metasilicate - Pentahydrate	212.7400	4.35	0.0204
Iron (III) Sulfate - Rhomboclase & Ferricopiapite	321.0340	3.90	0.0134
Magnesium Sulfate - Epsomite	246.4700	0.33	0.0013
**Fe:Mg ratio**	-	-	9:1

Solution Name	Molecular Weight (g/mol)	Amount (mL)	Amount Cation (mol)
Sodium Hydroxide (5 M)	-	19.80	-
H_2O	18.0153	417.11	-
Sulfuric Acid (0.5 M)	-	42.00	-
**Ending pH**	-	-	12.71

5-Mg 95-Fe^{3+}

Chemical Name	Molecular Weight (g/mol)	Amount (g or mL)	Amount (mol)
Sodium Metasilicate - Pentahydrate	212.7400	4.35	0.0204
Iron (III) Sulfate - Rhomboclase & Ferricopiapite	321.0340	4.25	0.0146
Magnesium Sulfate - Epsomite	246.4700	0.18	0.0007
**Fe:Mg ratio**	-	-	19:1

Solution Name	Molecular Weight (g/mol)	Amount (mL)	Amount Cation (mol)
Sodium Hydroxide (5 M)	-	19.80	-
H_2O	18.0153	417.11	-
Sulfuric Acid (0.5 M)	-	42.00	-
**Ending pH**	-	-	12.64

Supplementary Table 2: Concentration of reagent grade chemicals that were incorporated into solution to produce the synthesized products at 100° C

100-Fe Control

Chemical Name	Molecular Weight (g/mol)	Amount (g or mL)	Amount Cation (mol)
Sodium Metasilicate - Pentahydrate	212.7400	4.36	0.0205
Iron (II) Sulfate - Heptahydrate	278.0500	3.93	0.0141
Sodium Dithionite	174.1070	4.17	0.0240
**Fe:Mg ratio**	-	-	1:0

Solution Name	Molecular Weight (g/mol)	Amount (mL)	Amount Cation (mol)
Sodium Hydroxide (5 M)	NA	19.80	-
H_2O	18.0153	417.74	-
Sulfuric Acid (0.5 M)	NA	41.00	-
**Ending pH**	-	-	12.58

100-Fe^{3+}

Chemical Name	Molecular Weight (g/mol)	Amount (g or mL)	Amount (mol)
Sodium Metasilicate - Pentahydrate	212.7400	4.34	0.0204
Iron (III) Sulfate - Rhomboclase & Ferricopiapite	321.0340	4.04	0.0139
**Fe:Mg ratio**	-	-	1:0

Solution Name	Molecular Weight (g/mol)	Amount (mL)	Amount Cation (mol)
Sodium Hydroxide (5 M)	NA	19.80	-
H_2O	18.0153	417.87	-
Sulfuric Acid (0.5 M)	NA	35.00	-
**Ending pH**	-	-	12.87

100-Mg

Chemical Name	Molecular Weight (g/mol)	Amount (g or mL)	Amount (mol)
Sodium Metasilicate - Pentahydrate	212.7400	4.34	0.0204
Magnesium Sulfate - Epsomite	246.4700	3.51	0.0142
**Fe:Mg ratio**	-	-	0:1

Solution Name	Molecular Weight (g/mol)	Amount (mL)	Amount Cation (mol)
Sodium Hydroxide (5 M)	-	19.80	-
H_2O	18.0153	417.62	-
Sulfuric Acid (0.5 M)	-	35.00	-
**Ending pH**	-	-	12.98

15-Mg 85-Fe^{3+}

Chemical Name	Molecular Weight (g/mol)	Amount (g or mL)	Amount (mol)
Sodium Metasilicate - Pentahydrate	212.7400	4.34	0.0204
Iron (III) Sulfate - Rhomboclase & Ferricopiapite	321.0340	3.41	0.0117
Magnesium Sulfate - Epsomite	246.4700	0.50	0.0020
**Fe:Mg ratio**	-	-	17:3

Solution Name	Molecular Weight (g/mol)	Amount (mL)	Amount Cation (mol)
Sodium Hydroxide (5 M)	-	19.80	-
H_2O	18.0153	417.47	-
Sulfuric Acid (0.5 M)	-	35.00	-
**Ending pH**	-	-	12.89

50-Mg 50-Fe^{3+}

Chemical Name	Molecular Weight (g/mol)	Amount (g or mL)	Amount (mol)
Actual Experiment			
Sodium Metasilicate - Pentahydrate	212.7400	4.33	0.0204
Iron (III) Sulfate - Rhomboclase & Ferricopiapite	321.0340	2.00	0.0069
Magnesium Sulfate - Epsomite	246.4700	1.76	0.0071
**Fe:Mg ratio**	-	-	1:1

Solution Name	Molecular Weight (g/mol)	Amount (mL)	Amount Cation (mol)
Sodium Hydroxide (5 M)	-	19.80	-
H_2O	18.0153	416.62	-
Sulfuric Acid (0.5 M)	-	35.00	-
**Ending pH**	-	-	12.94

Supplementary Table 3: Mössbauer parameters for synthesized materials precipitated at 150° C, as well as natural nontronites for comparison.

Sample	Site	Weight	δ (Isomer Shift)	Δ (Quadrupole Splitting)	χ^2	Source
*Nontronite NAu-1	Fe^{3+}	0.61	0.35	0.24	2.10	This study
	Fe^{3+}	0.39	0.60	0.68		
*Experiment 1 (100-Fe Control - Subsequently Oxidized)	Fe^{3+}	0.60	0.81	1.09	5.57	This study
	Fe^{3+}	0.40	0.67	0.46		
*Experiment 4 (15-Mg 85-Fe^{3+})	Fe^{3+}	0.60	0.45	0.24	4.63	This study
	Fe^{3+}	0.40	0.64	0.68		
*Experiment 5 (50-Mg 50-Fe3+)	Fe^{3+}	0.54	0.33	0.24	2.35	This study
	Fe^{3+}	0.46	0.58	0.68		
Nontronite (Garfield)	Fe^{3+}	0.55	0.48	0.23	2.03	Ribeiro et al., 2009
	Fe^{3+}	0.43	0.47	0.65		
Nontronite	Fe^{3+}	NA	0.36-0.39	0.24-0.27	NA	Vandenberghe and De Grave, 2013
	Fe^{3+}	NA	0.37-0.40	0.59-0.68		
Nontronite (Washington)	Fe^{3+}	0.7	0.48	0.29	485	Goodman et al., 1976
	Fe^{3+}	0.24	0.48	0.62		
	Fe^{3+}_{tet}	0.06	0.29	0.6		
Nontronite (Washington)	Fe^{3+}	0.52	0.47	0.27	675	Goodman et al., 1976
	Fe^{3+}	0.48	0.45	0.62		
Nontronite (Garfield)	Fe^{3+}	0.54	0.5	0.27	437	Goodman et al., 1976
	Fe^{3+}	0.37	0.5	0.62		
	Fe^{3+}_{tet}	0.09	0.3	0.47		
Nontronite (Garfield)	Fe^{3+}	0.59	0.48	0.25	567	Goodman et al., 1976
	Fe^{3+}	0.41	0.47	0.65		
Nontronite (Clausthal)	Fe^{3+}	0.64	0.5	0.33	636	Goodman et al., 1976
	Fe^{3+}	0.21	0.5	0.67		
	Fe^{3+}_{tet}	0.15	0.3	0.61		
Nontronite (Crocidolite)	Fe^{3+}	0.59	0.49	0.34	469	Goodman et al., 1976
	Fe^{3+}	0.22	0.49	0.67		
	Fe^{3+}_{tet}	0.19	0.29	0.56		
Nontronite (Koegas)	Fe^{3+}	0.54	0.5	0.32	541	Goodman et al., 1976
	Fe^{3+}	0.19	0.51	0.62		
	Fe^{3+}_{tet}	0.27	0.31	0.53		
Nontronite (Amosite)	Fe^{3+}	0.51	0.49	0.34	556	Goodman et al., 1976
	Fe^{3+}	0.21	0.5	0.64		
	Fe^{3+}_{tet}	0.28	0.31	0.54		
Nontronite (California)	Fe^{3+}	0.44	0.5	0.33	475	Goodman et al., 1976
	Fe^{3+}	0.24	0.5	0.6		
	Fe^{3+}_{tet}	0.32	0.31	0.48		
Nontronite (Riverside)	Fe^{3+}	1	0.19	0.3	NA	Weldon et al., 1982
Nontronite (Washington)	Fe^{3+}	1	0.5	0.4	NA	Taylor et al., 1968

*Isomer shifts are reported relative to alpha-Fe.

Supplementary Table 4: Compositions of synthesized material determined through microprobe analysis

Weight Percent	0 - Mg Control	σ	0 - Mg	σ	100 - Mg	σ	15 - Mg 85 - Fe^{3+}	σ	50 -Mg 50 Fe^{3+}	σ
**Na_2O**	2.974	1.168	5.553	1.239	1.766	0.601	4.083	1.081	3.041	0.583
**Cr_2O_3**	0.011	0.009	0.010	0.015	0.017	0.022	0.012	0.016	0.019	0.020
**Cl**	0.022	0.016	0.031	0.012	0.024	0.012	0.018	0.016	0.014	0.014
**M_gO**	0.012	0.011	0.067	0.023	25.633	9.036	4.789	0.998	17.384	0.634
**SiO_2**	39.066	1.097	23.578	0.359	42.078	3.866	32.060	2.033	41.958	1.063
**TiO_2**	0.021	0.026	0.015	0.017	0.015	0.014	0.024	0.020	0.015	0.019
**K_2O**	0.399	0.135	0.656	0.130	0.208	0.041	0.627	0.263	0.383	0.162
**Al_2O_3**	0.078	0.021	0.144	0.050	0.090	0.059	0.117	0.046	0.113	0.033
**MnO**	0.035	0.017	0.034	0.021	0.014	0.015	0.041	0.024	0.033	0.024
**CaO**	0.042	0.030	0.117	0.030	0.039	0.014	0.070	0.027	0.054	0.022
**F**	0.020	0.057	0.000	0.000	0.074	0.097	0.018	0.036	0.011	0.023
**FeO**	48.383	1.286	65.221	1.068	0.052	0.027	54.699	2.549	31.758	0.981
**P_2O_5**	0.452	0.133	1.464	0.272	0.277	0.198	0.547	0.151	0.406	0.189
Total	91.502	1.315	96.881	2.148	70.251	11.770	97.094	2.050	95.181	1.129

*Low totals, characteristic of many clay minerals, are likely due to dehydration under desiccation and significant structural OH. In addition, EMP analysis relies on a relatively flat sample surfaces; this can be difficult to achieve, due to etching and pitting during the polishing process. Clay minerals are also highly porous, which may contribute to low totals observed in this study and within the literature by EMP analysis[17].

Supplementary Table 5: Composition of synthesized materials determined through Inductively Coupled Plasma – Optical Emission Spectroscopy.

	Nau-1	0 - Mg Control	0 - Mg	100 - Mg	15 - Mg	50 - Mg	5 - Mg
Al2O3	13.92	0.05	0.05	0.08	0.04	0.16	0.00
CaO	2.08	0.00	0.17	0.04	0.12	0.19	0.00
FeOT	29.22	56.23	62.73	0.03	49.39	30.48	53.03
K2O	0.22	0.02	0.00	0.00	0.00	0.00	0.00
MgO	1.15	0.01	0.05	33.50	5.16	17.19	1.46
Na2O	0.22	4.88	10.72	12.29	13.74	9.55	16.33
SiO2	53.18	38.82	26.28	54.05	31.56	42.44	29.17

*A conservative upper limit on the uncertainty in the chemical analysis is 10 percent.

Supplementary Table 6: Proposed chemical formulae for select experiments, and the calculated amount of Fe and Mg secondary phases.

Experiment	Formula	Volume (cm^3)	% Mass	Moles Fe-Residual	Volume (cm^3)	% Mass	Moles Mg-Residual	Volume (cm^3)	% Mass
100-Fe Control	$Na_{0.75}[Fe^{3+}{}_{2}][Si_{3.25}Fe^{3+}{}_{0.75}]O_{10}(OH)_2$	132.11	78.2	$1.19Fe(OH)_3$	40.8884	21.8	-	0	0.0
100-Fe^{3+}	$Na_{0.75}[Fe^{3+}{}_{2}][Si_{3.25}Fe^{3+}{}_{0.75}]O_{10}(OH)_2$	132.11	53.3	$3.74Fe(OH)_3$	128.5064	46.7	-	0	0.0
5-Mg 95-Fe^{3+}	$Na_{0.75}[Fe^{3+}{}_{1.91}Mg_{0.13}][Si_{3.25}Fe^{3+}{}_{0.75}]O_{10}(OH)_2$	132.32	63.7	$2.28Fe(OH)_3$	78.3408	35.4	$0.11Mg(OH)_2$	2.7093	0.9
15-Mg 85-Fe^{3+}	$Na_{0.75}[Fe^{3+}{}_{1.69}Mg_{0.46}][Si_{3.25}Fe^{3+}{}_{0.75}]O_{10}(OH)_2$	132.84	67.8	1.81Fe(OH)3	62.1916	29.2	0.34Mg(OH)2	8.3742	3.0
50-Mg 50-Fe^{3+}	$Na_{0.25}[Fe^{3+}{}_{1.09}Mg_{1.36}][Si_{3.75}Fe^{3+}{}_{0.25}]O_{10}(OH)_2$	134.42	73.2	$0.91Fe(OH)_3$	31.2676	17.2	$0.92Mg(OH)_2$	22.6596	9.5
100-Mg	$Na_{0.25}[Mg_{2.75}][Si_4]O_{10}(OH)_2$	136.69	86.7	-	0	0.0	$0.97Mg(OH)_2$	23.8911	13.3

To generate mineral percentages we assume that the interlayer charge is 0.25 (for a $O_{10}(OH)_2$ half unit cell) for all samples that did expand to the classic ~17 Å upon glycolation (100-Mg and the 50:50 Mg:Fe). The value of 0.75 is used for samples that did not expand to ~17 Å upon glycolation[2]. Fe and Mg were added at the ratio determined through ICP-OES to the octahedral and tetrahedral sites to balance the required charge. Because Mg does not reside within tetrahedral sites[18], Mg was given priority when assigning it to the octahedral sites. The remaining Fe not present in the octahedral layer was then placed within the tetrahedral sites. The remaining Fe and Mg not assigned to the clay mineral structure was portioned into the secondary phases observed by µXRD (e.g. ferrihydrite and/or brucite). The resulting Fe:Mg ratio of the bulk clay minerals is within one percent (molar ratio) of the initial solution chemistry. The lower ratios of Si to Fe and Mg may have resulted from the incomplete solubilization of Si, as HF was not added during the digestion[19]. The Mg:Fe ratio in the octahedral layer determined using the assumptions described above was used to define the tri- and di-octahedral nature of the precipitates.

Supplementary Table 7: Compositions of naturally occurring nontronites, all of which contain at least minor concentrations of Mg.

Name	Formula	Source
NAU-1 - Nontronite	$M^{2+}_{0.5025}[Al_{1.02}Si_{6.98}][Fe_{3.68}Mg_{0.04}Al_{0.29}]O_{20}(OH)_4$	Keeling et al., 2000
NAU-2 - Nonronite	$M^{2+}_{0.36}[Al_{0.45}Si_{7.55}][Fe_{3.83}Mg_{0.05}]O_{20}(OH)_4$	Keeling et al., 2000
Garfield - Nontronite	$M^{2+}_{0.405}[Al_{0.780}Si_{7.22}][Al_{0.31}Fe^{3+}_{3.64}Fe^{2+}_{0.01}Mg_{0.04}]O_{20}(OH)_4$	Manceau et al., 2000
PV - Nontronite	$Mg_{0.445}[Al_{0.43}Si_{7.57}][Al_{0.65}Fe^{3+}_{2.87}Fe^{2+}_{0.01}Mg_{0.47}]O_{20}(OH)_4$	Manceau et al., 2000
SWA-1 - Nontronite	$M^{2+}_{0.435}[Al_{0.623}Si_{7.38}][Al_{1.08}Fe^{3+}_{2.67}Fe^{2+}_{0.01}Mg_{0.23}]O_{20}(OH)_4$	Manceau et al., 2000
NG-1 - Notnronite	$M^{2+}_{0.35}[Fe^{3+}_{0.63}Al_{0.08}Si_{7.29}][Al_{0.88}Fe^{3+}_{3.08}Fe^{2+}_{0.01}Mg_{0.06}]O_{20}(OH)_4$	Manceau et al., 2000
Chad - Nontronite	$M^{2+}_{0.22}[Al_{0.17}Si_{3.83}][Fe_{1.77}Mg_{0.21}]O_{10}(OH)_2$	Tardy and Frtiz, 1981
Caledonia - Nontornite	$M^{2+}_{0.135}[Al_{0.27}Si_{3.73}][Al_{0.07}Fe_{1.72}Mg_{0.315}]O_{10}(OH)_2$	Tardy and Fritz, 1981
CLA - Nontornite	$M^{2+}_{0.52}[Al_{0.13}Si_{6.81}Fe_{1.06}][Fe_{4.01}Mg_{0.07}]O_{20}(OH)_4$	Goodman et al., 1976
CRO - Nontronite	$M^{2+}_{0.535}[Al_{0.063}Si_{6.75}Fe_{1.19}][Fe_{3.90}Mg_{0.24}]O_{20}(OH)_4$	Goodman et al., 1976
CAL - Nontornite	$M^{2+}_{0.625}[Al_{0.14}Si_{6.21}Fe_{1.65}][Fe_{4.04}Mg_{0.21}]O_{20}(OH)_4$	Goodman et al., 1976
AMO - Nontronite	$M^{2+}_{0.37}[Al_{0.04}Si_{6.84}Fe_{1.12}][Fe_{4.04}Mg_{0.15}]O_{20}(OH)_4$	Goodman et al., 1976
GAR - Nontronite	$M^{2+}_{0.6}[Al_{1.05}Si_{6.84}Fe_{0.11}][Fe_{3.96}Mg_{0.04}]O_{20}(OH)_4$	Goodman et al., 1976
WAS - Nontornite	$M^{2+}_{0.405}[Al_{0.70}Si_{7.30}][Al_{1.06}Fe_{2.73}Mg_{0.26}]O_{20}(OH)_4$	Goodman et al., 1976
KOE - Nontronite	$M^{2+}_{0.505}[Al_{0.08}Si_{6.61}Fe_{1.31}][Fe_{4.06}Mg_{0.10}]O_{20}(OH)_4$	Goodman et al., 1976
Fe-Smectite	$M^{2+}_{0.475}[Al_{0.42}Si_{7.58}][Al_{1.49}Fe_{2.07}Mg_{0.39}]O_{20}(OH)_4$	Gates et al., 2002
SWa-1	$M^{2+}_{0.475}[Al_{0.60}Si_{7.40}][Al_{1.10}Fe_{2.62}Mg_{0.25}]O_{20}(OH)_4$	Gates et al., 2002
Cheney	$M^{2+}_{0.500}[Al_{0.94}Si_{7.06}][Al_{1.00}Fe_{2.95}Mg_{0.05}]O_{20}(OH)_4$	Gates et al., 2002
Giralong	$M^{2+}_{0.460}[Al_{0.63}Fe_{0.05}Si_{7.32}][Al_{0.44}Fe_{3.29}Mg_{0.28}]O_{20}(OH)_4$	Gates et al., 2002
Manito	$M^{2+}_{0.510}[Al_{0.81}Fe_{0.12}Si_{7.07}][Al_{0.42}Fe_{3.47}Mg_{0.28}]O_{20}(OH)_4$	Gates et al., 2002
NAu-1	$M^{2+}_{0.525}[Al_{0.81}Fe_{0.07}Si_{6.98}][Al_{0.36}Fe_{3.61}Mg_{0.04}]O_{20}(OH)_4$	Gates et al., 2002
Bingham	$M^{2+}_{0.530}[Al_{0.59}Fe_{0.24}Si_{7.17}][Al_{0.30}Fe_{3.47}Mg_{0.23}]O_{20}(OH)_4$	Gates et al., 2002
Garfield	$M^{2+}_{0.535}[Al_{0.88}Fe_{0.10}Si_{7.02}][Al_{0.33}Fe_{3.63}Mg_{0.03}]O_{20}(OH)_4$	Gates et al., 2002
Mountainville	$M^{2+}_{0.520}[Al_{0.77}Fe_{0.22}Si_{7.01}][Al_{0.41}Fe_{3.54}Mg_{0.04}]O_{20}(OH)_4$	Gates et al., 2002
NG-1	$M^{2+}_{0.480}[Al_{0.26}Fe_{0.62}Si_{7.12}][Al_{0.72}Fe_{3.22}Mg_{0.05}]O_{20}(OH)_4$	Gates et al., 2002
NAu-1	$M^{2+}_{0.360}[Al_{0.16}Fe_{0.29}Si_{7.55}][Al_{0.52}Fe_{3.45}Mg_{0.03}]O_{20}(OH)_4$	Gates et al., 2002
HQ-Tasmania	$M^{2+}_{0.515}[Al_{0.60}Fe_{0.42}Si_{6.98}][Al_{0.52}Fe_{3.45}Mg_{0.03}]O_{20}(OH)_4$	Gates et al., 2002
Spokane	$M^{2+}_{0.460}[Al_{0.04}Fe_{0.70}Si_{7.26}][Al_{0.03}Fe_{3.89}Mg_{0.04}]O_{20}(OH)_4$	Gates et al., 2002
CZ-Germany	$M^{2+}_{0.500}[Al_{0.04}Fe_{0.92}Si_{704}][Al_{0.23}Fe_{3.73}Mg_{0.04}]O_{20}(OH)_4$	Gates et al., 2002

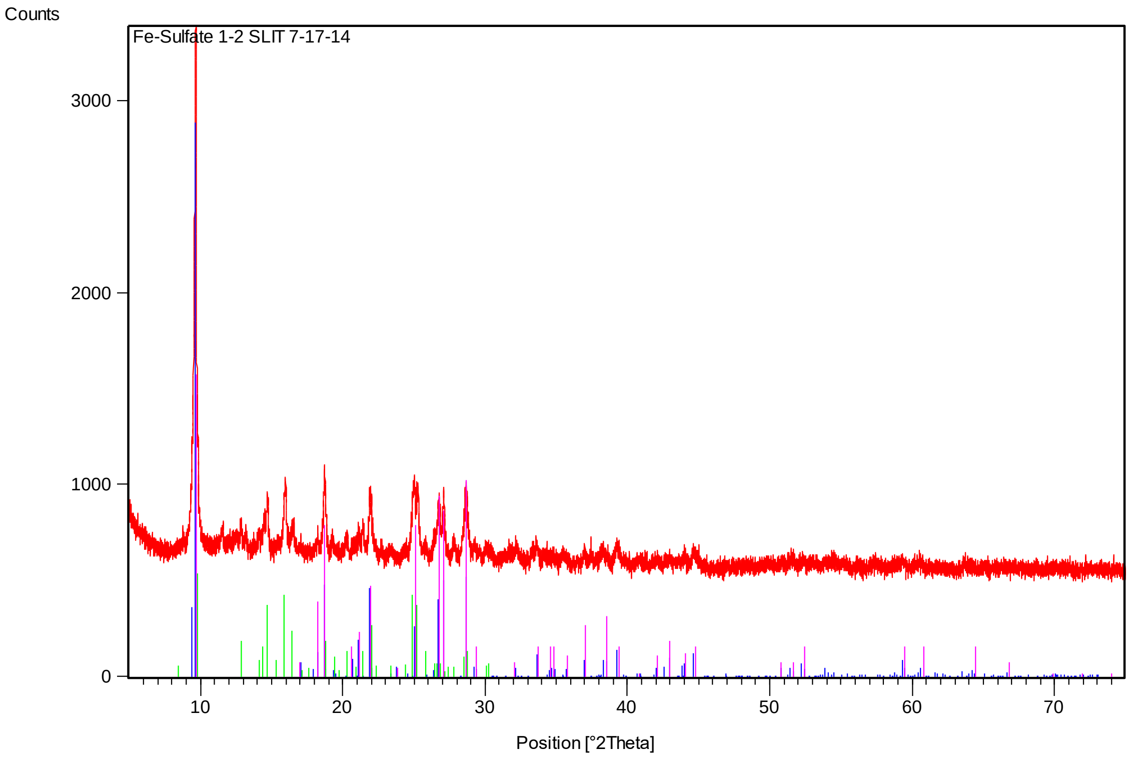

Visible	Ref. Code	Score	Compound Name	Displacement [°2Th.]	Scale Factor	Chemical Formula
*	01-070-1820	30	Rhomboclase, syn	0.000	0.851	(H5 O2) Fe (S O4)2 (H2 O)2
*	00-029-0714	29	Ferricopiapite	0.000	0.158	Fe4.67 (S O4)6 (O H)2 !20 H2 O
*	00-025-0421	19	Rhomboclase	0.000	0.465	H Fe (S O4)2 !4 H2 O

Supplementary Figure 1: Diffraction pattern of the reagent grade Fe^{3+}-sulfate. This diffraction pattern is indicative of the Fe-sulfates rhomboclase – $HFe^{3+}(SO_4)_2\cdot 4(H_2O)$ and ferricopiapite – $Fe^{3+}_{0.66}Fe^{3+}_4(SO_4)_6(OH)_2\cdot 20(H_2O)$.

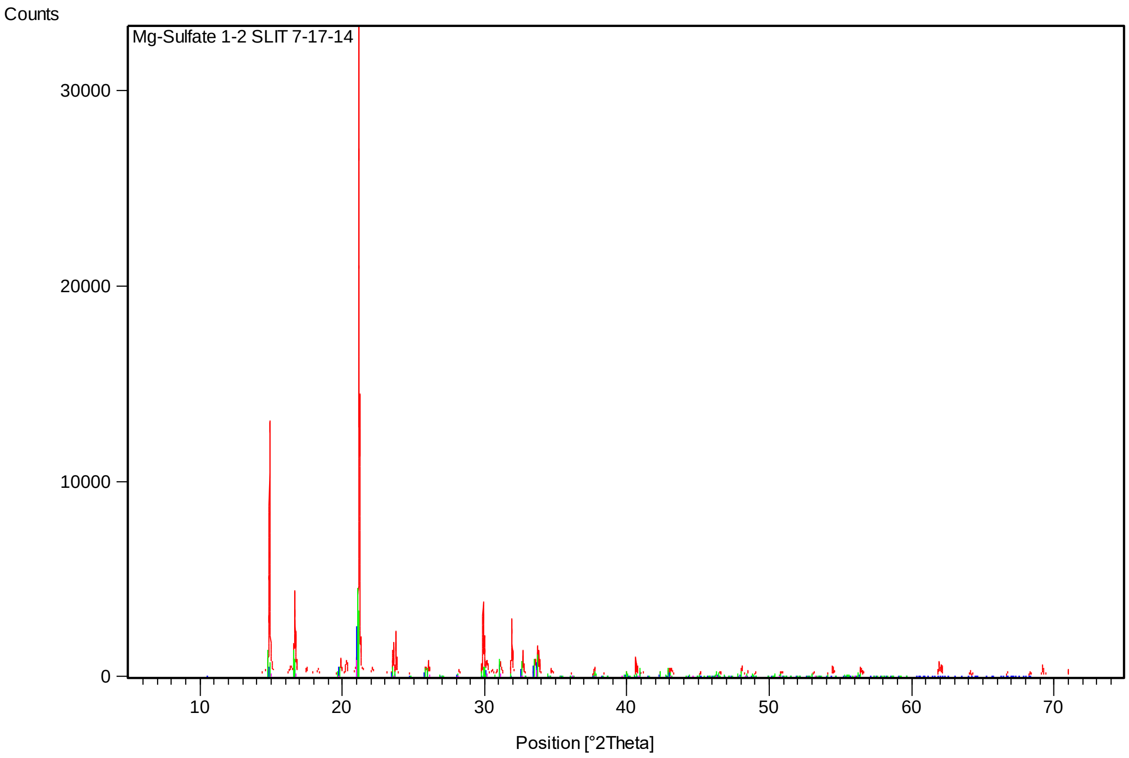

Visible	Ref. Code	Score	Compound Name	Displacement [°2Th.]	Scale Factor	Chemical Formula
*	01-072-0696	46	Epsomite, syn	0.000	0.081	Mg S O4 (H2 O)7
*	00-036-0419	39	Epsomite, syn	0.000	0.136	Mg S O4 !7 H2 O
*	00-001-0399	35	Epsomite	0.000	0.026	Mg S O4 !7 H2 O

Supplementary Figure 2: Diffraction pattern of the Mg-sulfate. This diffraction pattern is indicative of the Mg-sulfate epsomite – $MgSO_4{\cdot}7(H_2O)$.

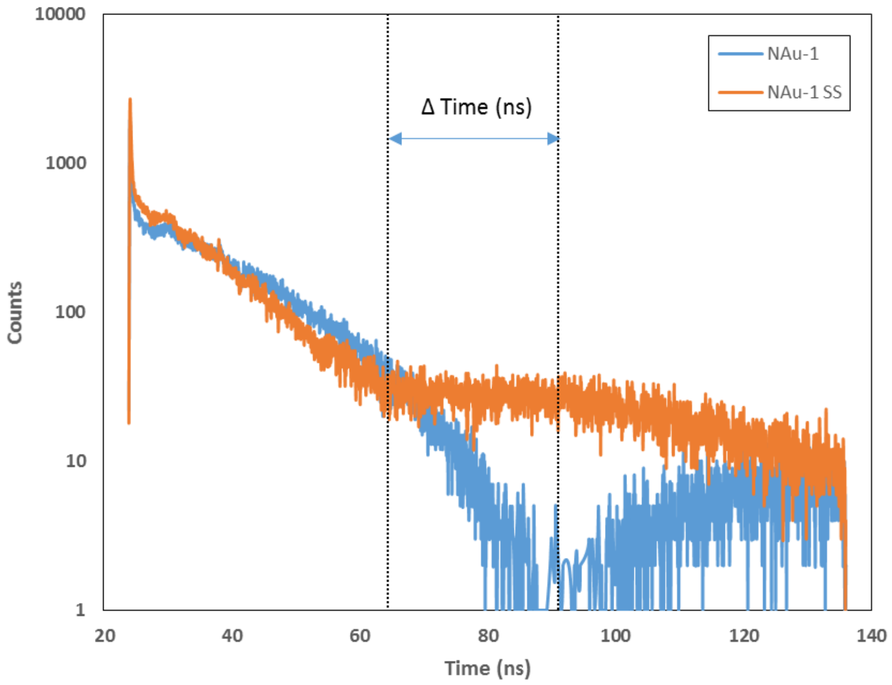

Supplementary Figure 3: Synchrotron Mössbauer Spectra of NAu-1 (blue) and NAu-1 with a 10μm stainless steel foil (orange) to determine center shifts (blue arrow). Note the increased abundance of ^{57}Fe results in a shift to lower time for the sample with stainless steel foil. The isomer shifts and quadrupole splittings of both Fe^{3+} sites occur within the single observed absorption which is consistent with the ferric-clay mineral nontronite[13-15].

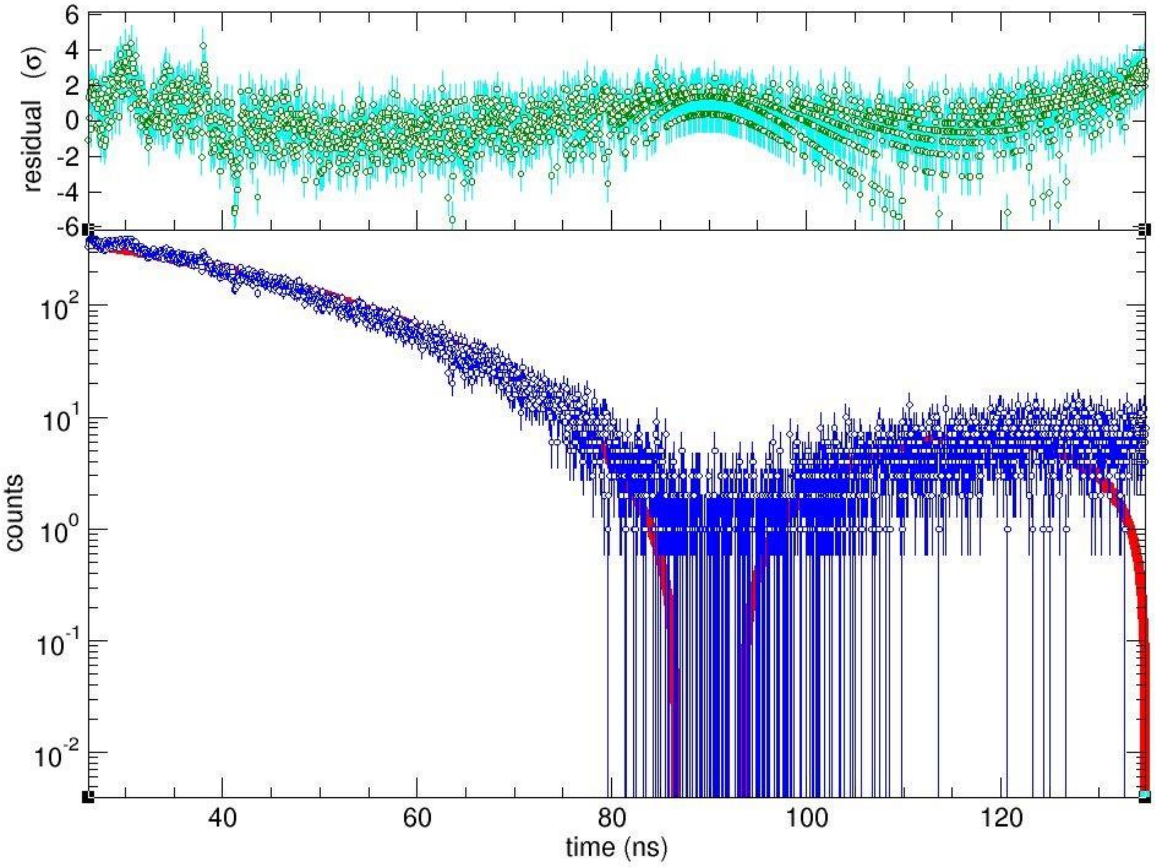

Supplementary Figure 4: Synchrotron Mössbauer data of NAu-1 without stainless steel foil. The plot was fitted in CONUSS[21]. Blue lines represent the error of the analysis.

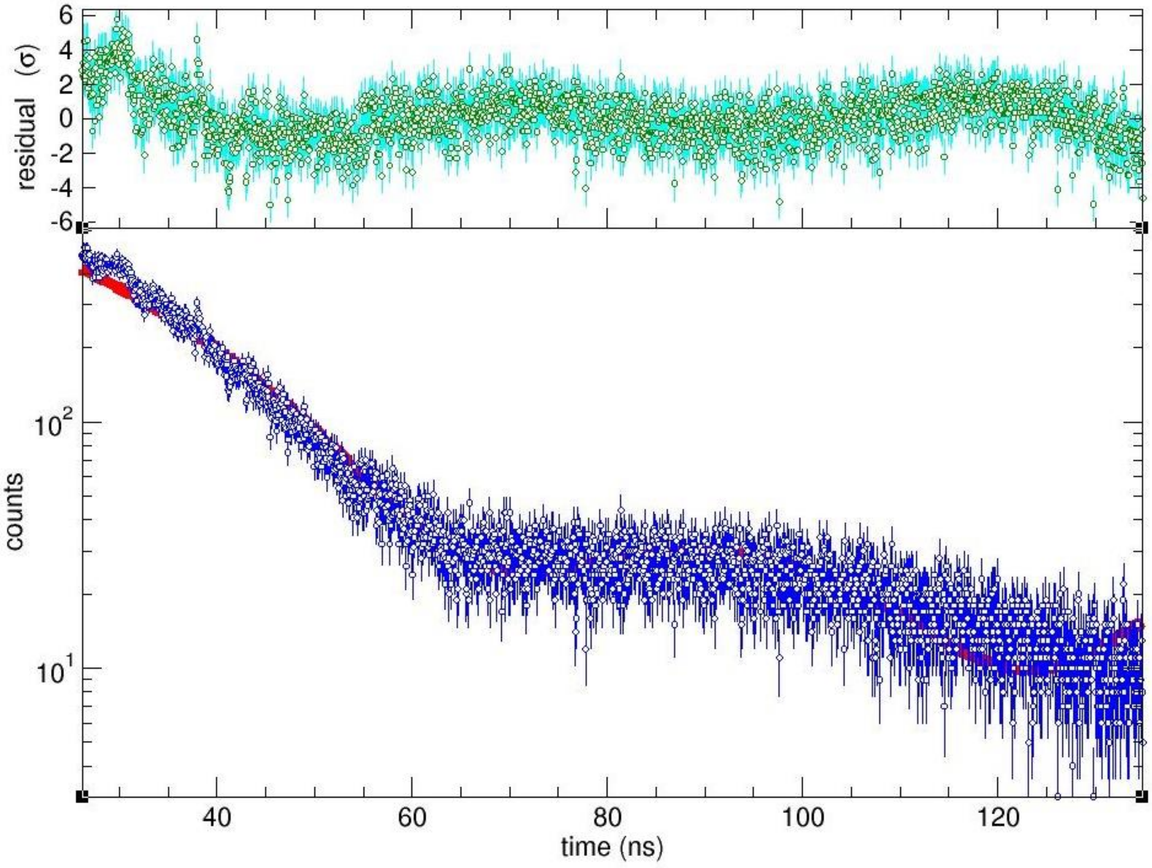

Supplementary Figure 5: Synchrotron Mössbauer data of NAu-1 with stainless steel foil. The plot was fitted in CONUSS[21]. Blue lines represent the error of the analysis.

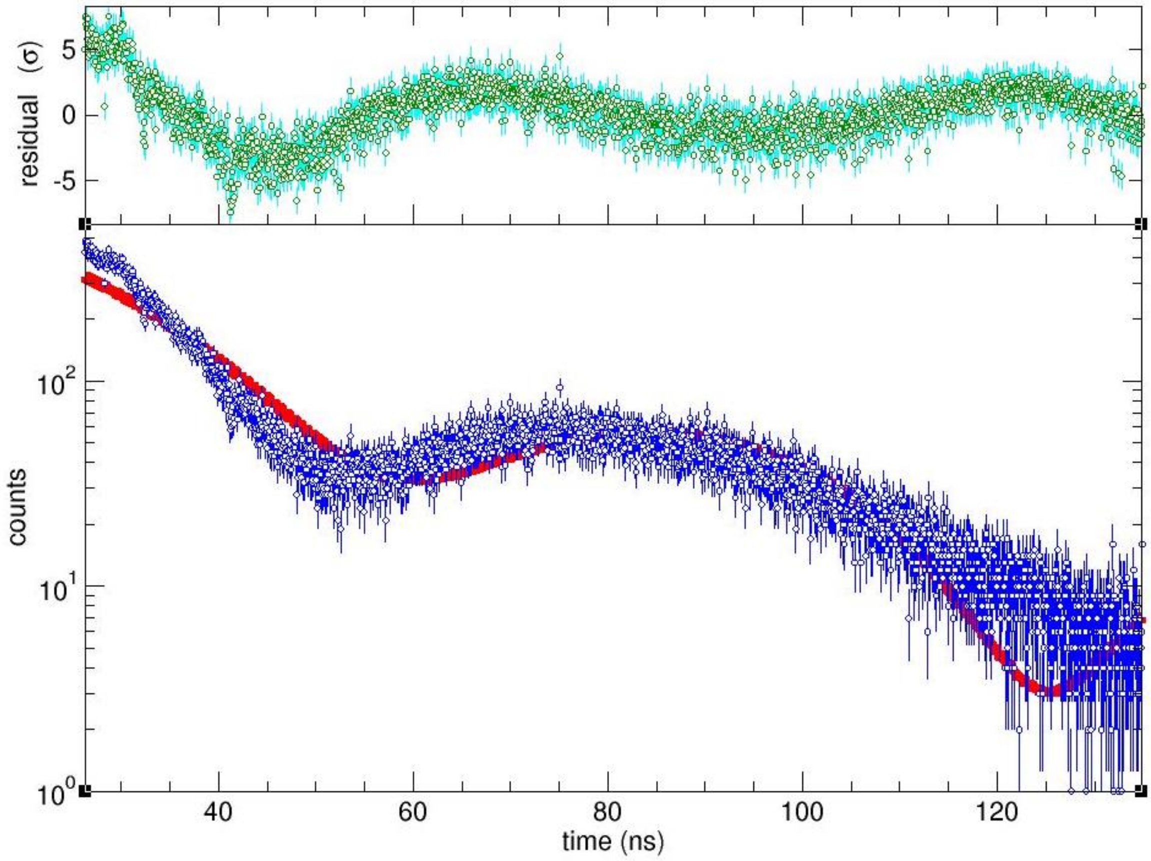

Supplementary Figure 6: Synchrotron Mössbauer data of the 15-Mg 85-Fe^{3+} precipitate (150º C) with stainless steel foil. The plot was fitted in CONUSS[21]. Blue lines represent the error of the analysis. The variation between the fit produced within CONUSS and the actual data is likely due to the low number of counts towards higher time. However, the very low number of counts in this time-range are a minor contribution to the total spectra.

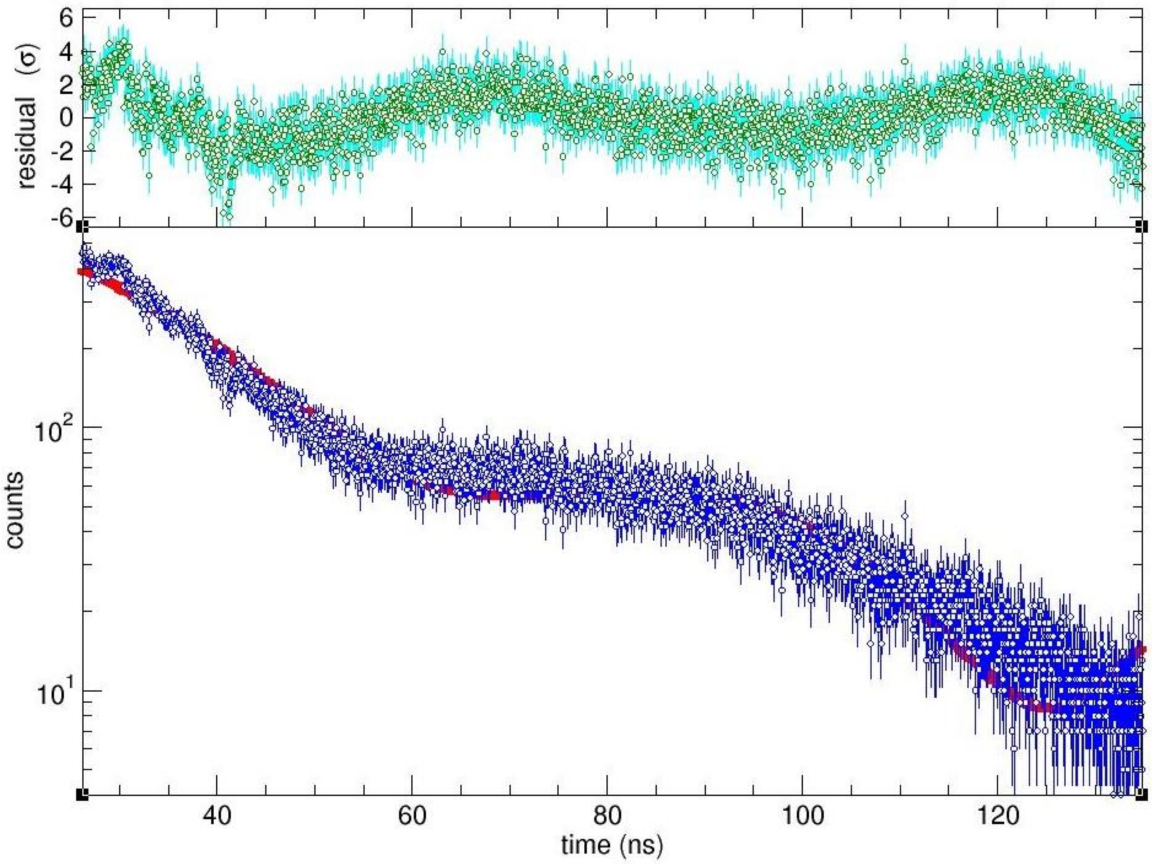

Supplementary Figure 7: Synchrotron Mössbauer data of the 50-Mg 50-Fe^{3+} precipitate (150º C) with stainless steel foil. The plot was fitted in CONUSS[21]. Blue lines represent the error of the analysis. The variation between the fit produced within CONUSS and the actual data is likely due to the low number of counts towards higher time. However, the very low number of counts in this time-range are a minor contribution to the total spectra.

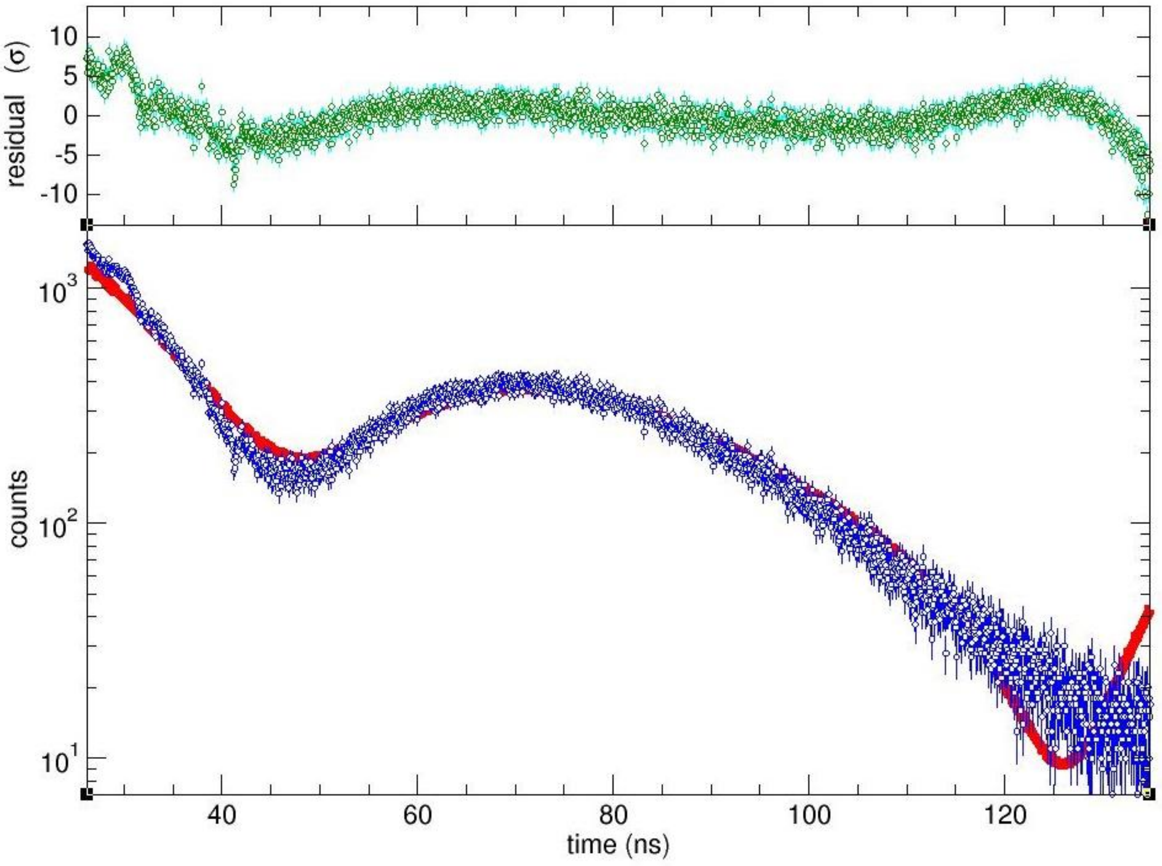

Supplementary Figure 8: Synchrotron Mössbauer data of the 100-Fe Control - Subsequently Oxidized precipitate (150º C) with stainless steel foil. The plot was fit in CONUSS[21]. Blue lines represent the error of the analysis. The variation between the fit produced within CONUSS and the actual data is likely due to the low number of counts towards higher time. However, the very low number of counts in this time-range are a minor contribution to the total spectra.

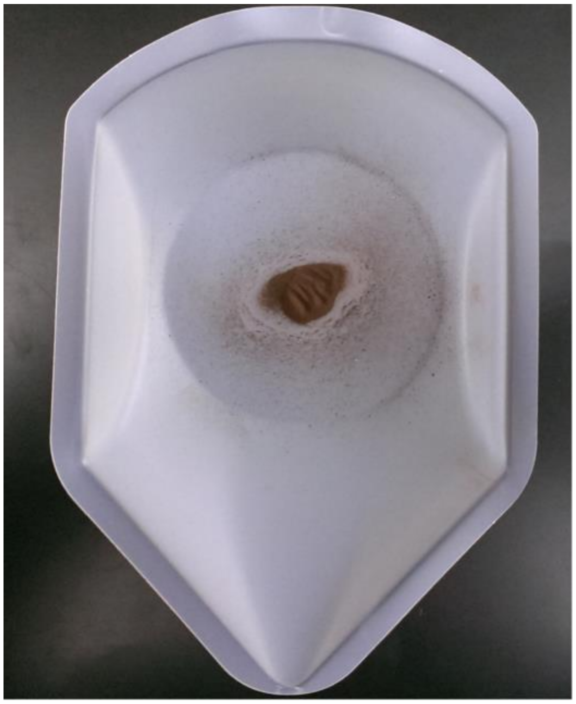

Supplementary Figure 9: Photograph of the synthetic nontronite control (100-Fe Control - Subsequently Oxidized) precipitated at 150º C, dried in-vacuo and ground to a fine powder in a mortar and pestle. The reddish color is likely due to the oxidation of the ferrous precursor into nontronite as indicated by our Mössbauer data (Table S3 and S8), as well as the Mössbauer spectroscopy data collected by Mizutani et al.[1] on material previously produced by this method.

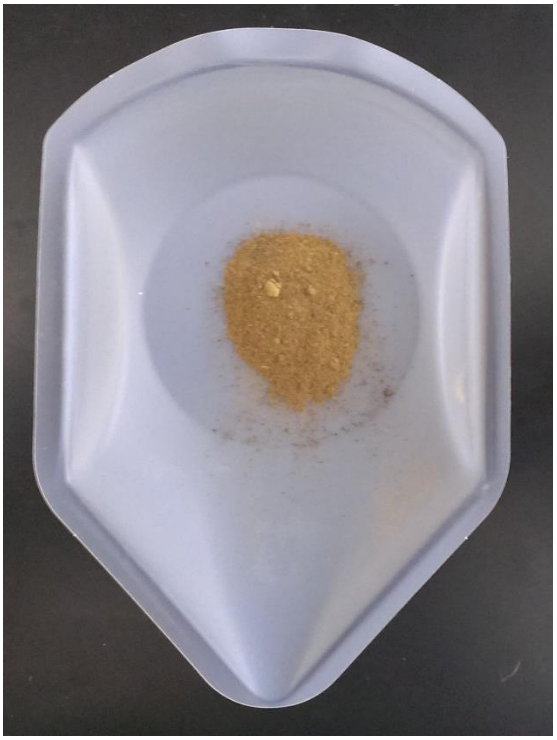

Supplementary Figure 10: Photograph of the synthetic nontronite control (100-Fe Control - Subsequently Oxidized) precipitated at 100º C, dried in-vacuo and ground to a fine powder in a mortar and pestle. The reddish color is likely due to the oxidation of the ferrous precursor into nontronite. This is supported by the Mössbauer spectroscopy data collected by Mizutani et al.[1] on material synthesized in an similar manner, as well as our Mössbauer data of the corresponding 150º C experiment (Table S3 and Figure S8).

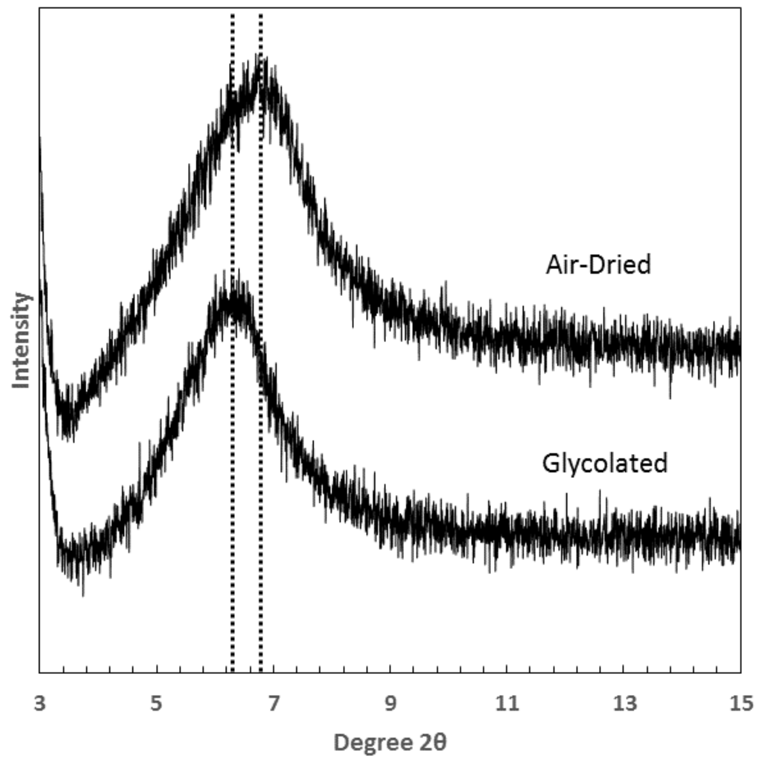

Supplementary Figure 11: X-Ray diffraction pattern of the 100-Fe Control - Subsequently Oxidized, precipitated at 150º C. Note the slight expansion when exposed to ethylene glycol vapor for over 24 hours. The clay mineral did not expand to the diagnostic 17 Å of smectite, but only to approximately 14 Å which is consistent with previous descriptions of material synthesized in this manner as a high-charge (expands very little during exposure to ethylene glycol vapor) nontronite[2].

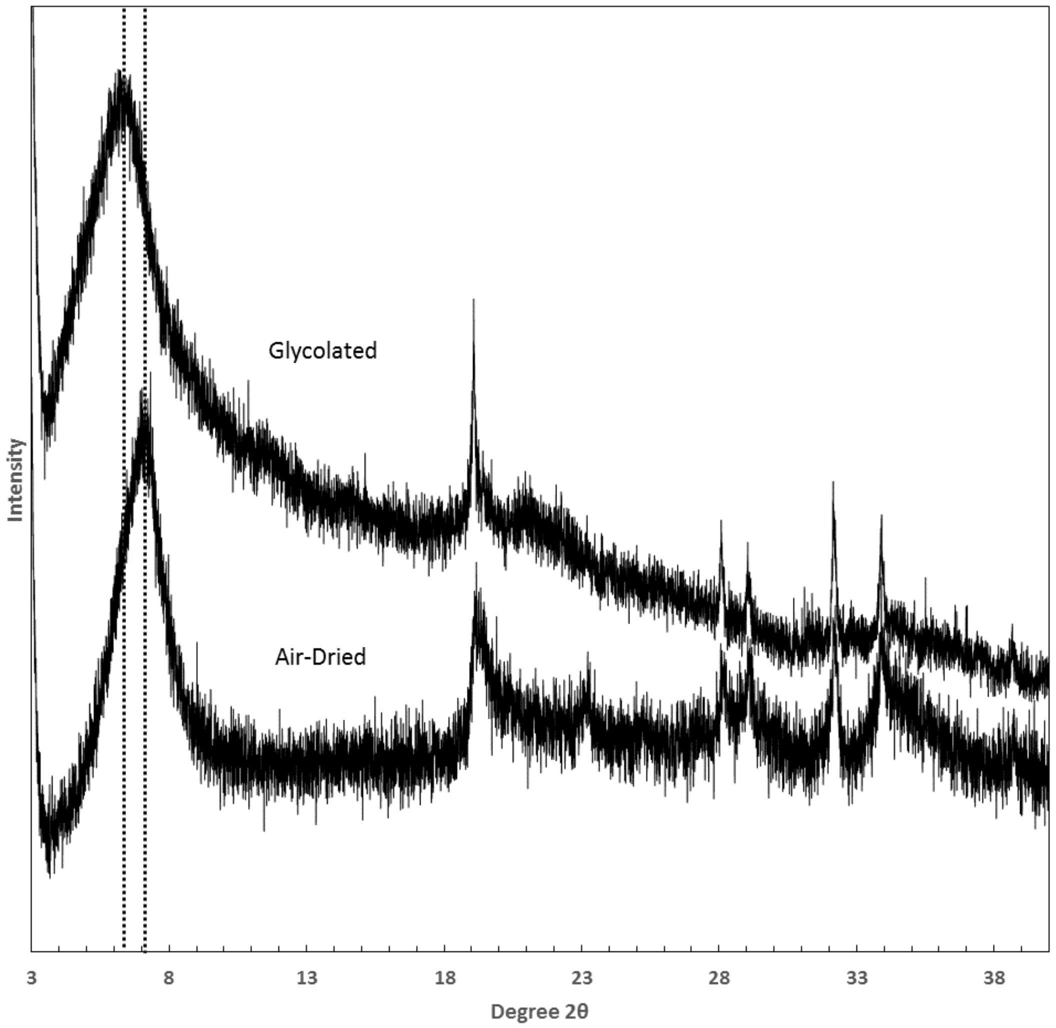

Supplementary Figure 12: X-Ray diffraction pattern of the 100-Fe Control - Subsequently Oxidized precipitated at 100º C - note the low angle broad reflection, which is indicative of clay minerals. In addition, the diffractions occurring at approximately 20 degrees (020 reflection) characterizes the b dimension of the unit cell and is also indicative of clay minerals, having been used to identify clay mineralogy on Mars[17]. Material synthesized in this manner has been previously characterized as high-charge (e.g. expands very little when exposed to ethylene glycol vapor) nontronite[2].

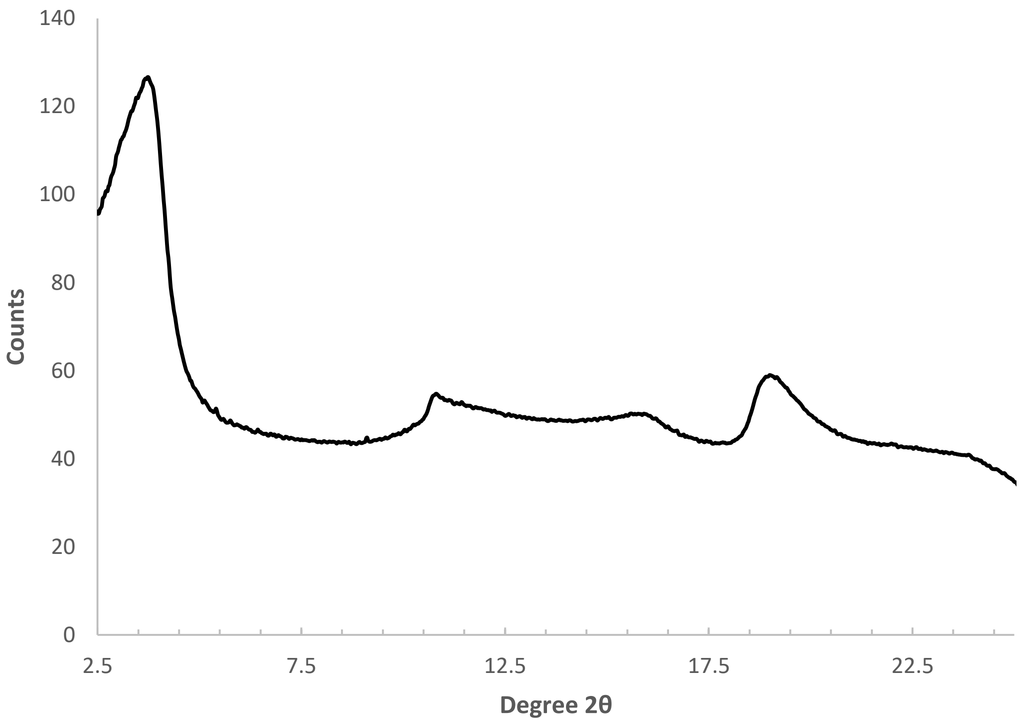

Supplementary Figure 13: Synchrotron μ-XRD (0.86 Å wavelength) pattern for the 100-Fe Control - Subsequently Oxidized) precipitated at 150º C. Note the multiple peaks produced which are the direct result of crystallinity. This diffraction pattern is consistent with a clay mineral structure in that the diffraction peaks are produced through the 001 (large low-angle reflection) 020 and 060 reflections.

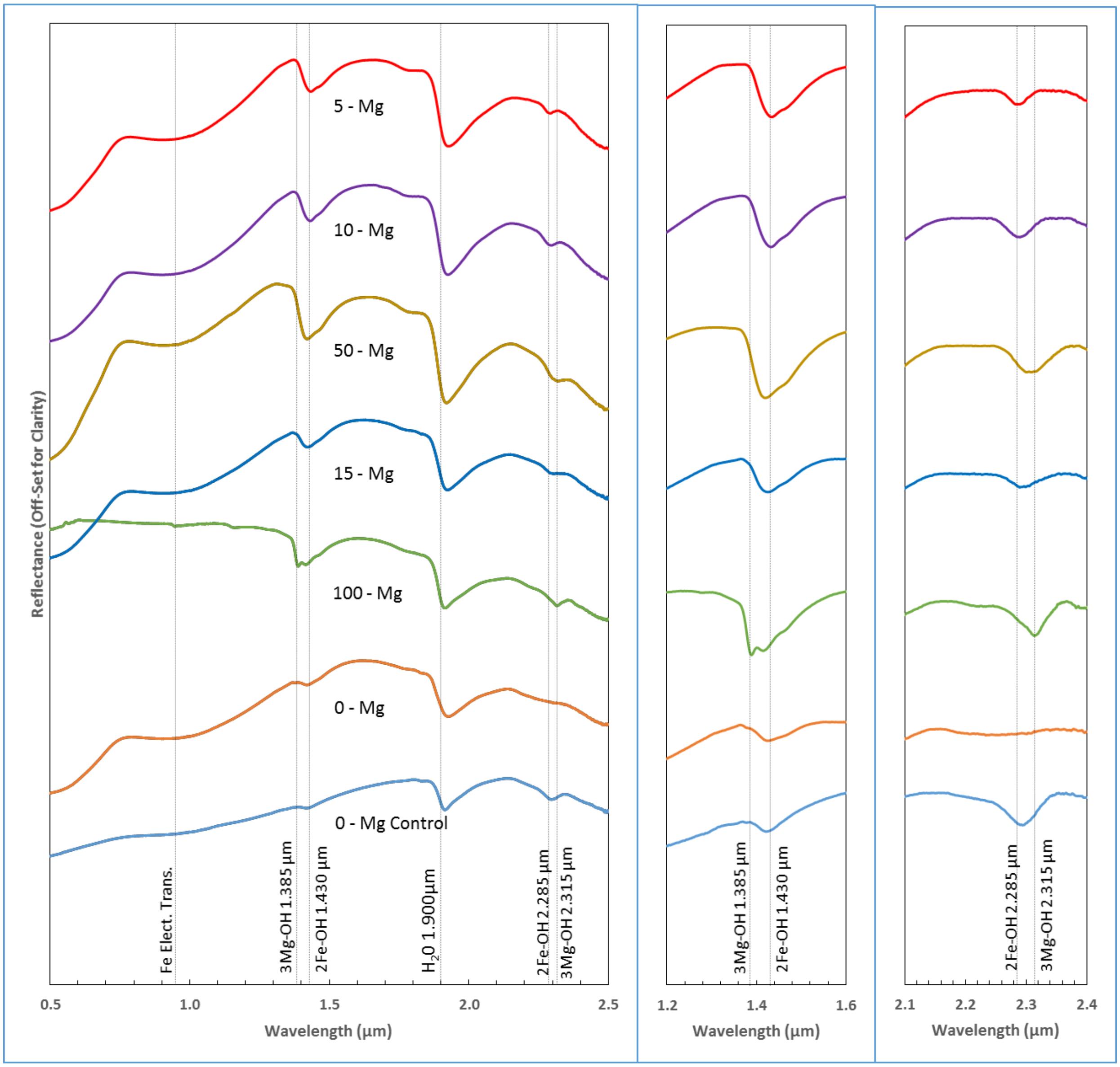

Supplementary Figure 14: Visible Near Infrared (VNIR) spectra of the synthesized clay minerals precipitated at 150º C as described in the methods. For complete information regarding the compositions see Table 1. Spectra were collected with an Analytical Spectra Devices (ASD) VNIR spectrometer. The continuum has been removed in the right columns. 0-Mg-control refers to the sample synthesized with initial Fe^{2+}.

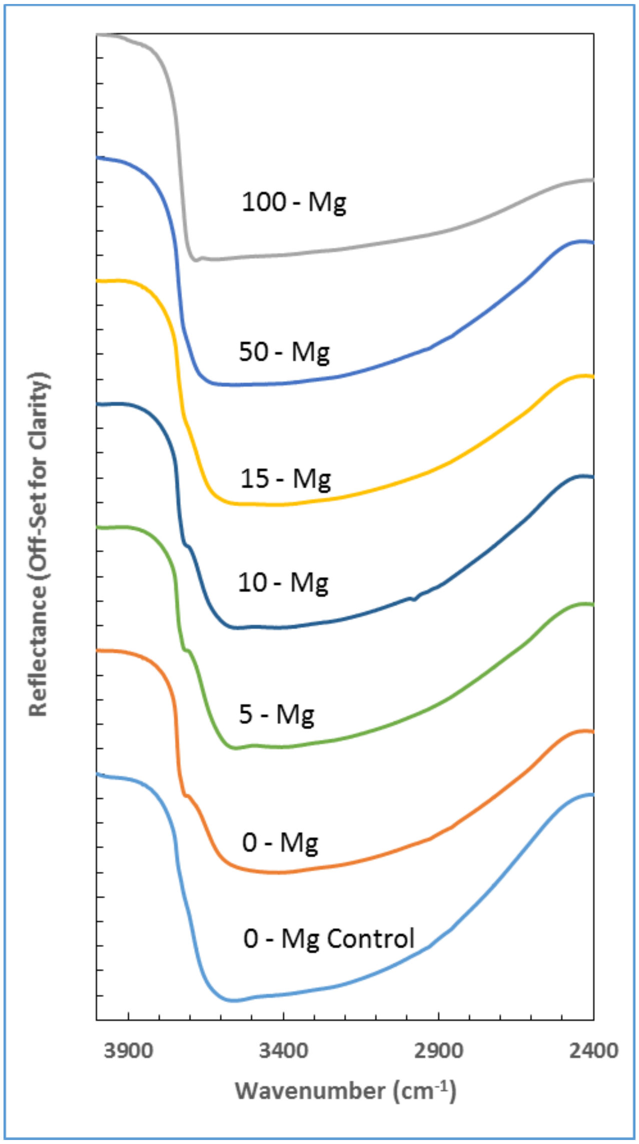

Supplementary Figure 15: Spectra of the 4000 to 2400 cm^{-1} region, for the 150° C precipitates. Note the systematic shift of absorptions to higher energies (lower wavenumber) with increasing Mg, with the 3Mg-OH absorption occurring at 2.72 μm (3676 cm^{-1}) in Mg-rich clay minerals. The 2Fe-OH absorption occurs at a lower wavelength of 2.80 μm (3571 cm^{-1}). These spectra have absorptions characteristic of Fe and/or Mg-rich clay minerals.

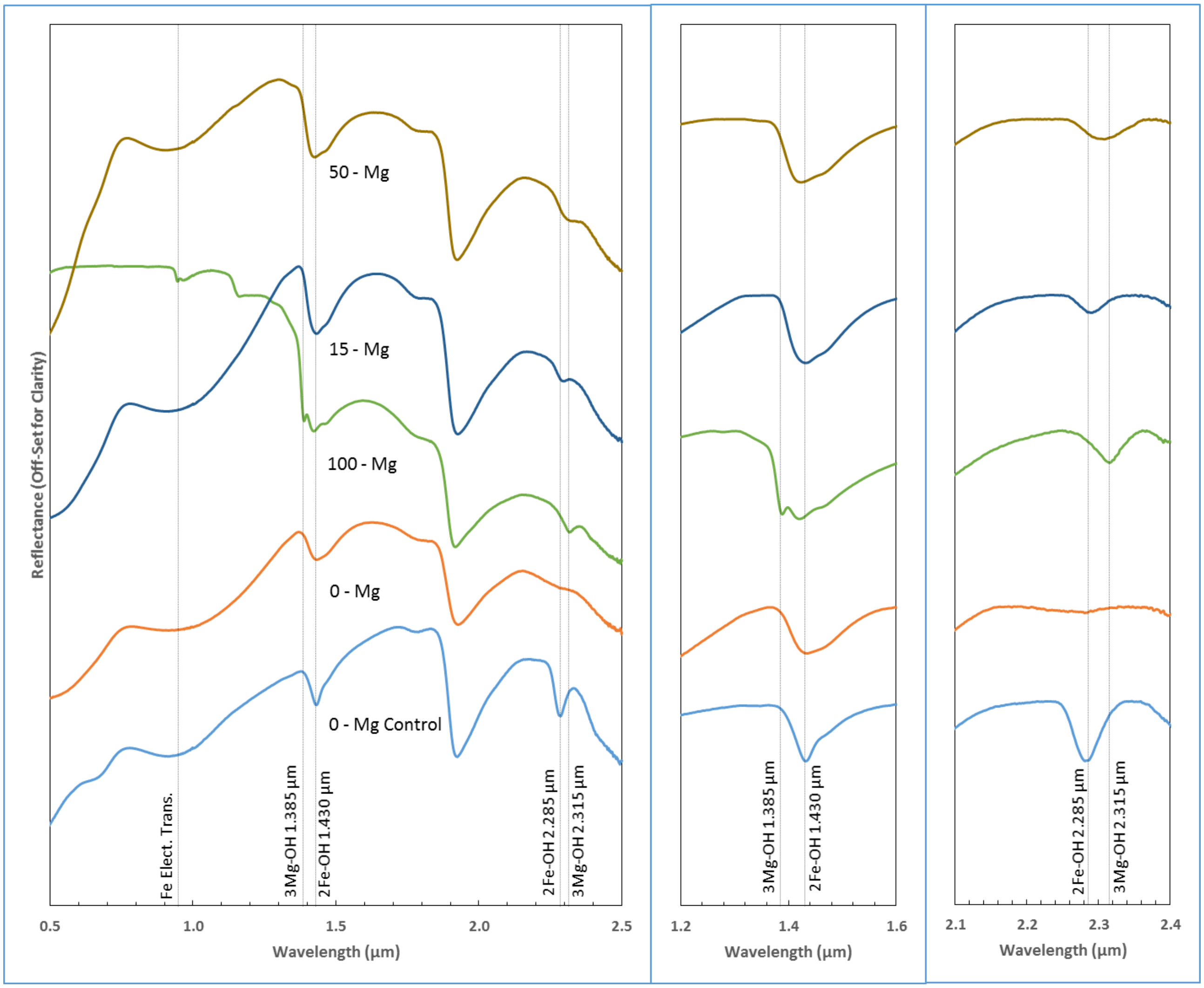

Supplementary Figure 16: Visible Near Infrared (VNIR) spectra of the synthesized clay minerals precipitated at 100° C as described in the methods. For complete information regarding the compositions see Table 2. Spectra were collected with an Analytical Spectra Devices (ASD) VNIR spectrometer. The continuum has been removed in the right two columns.

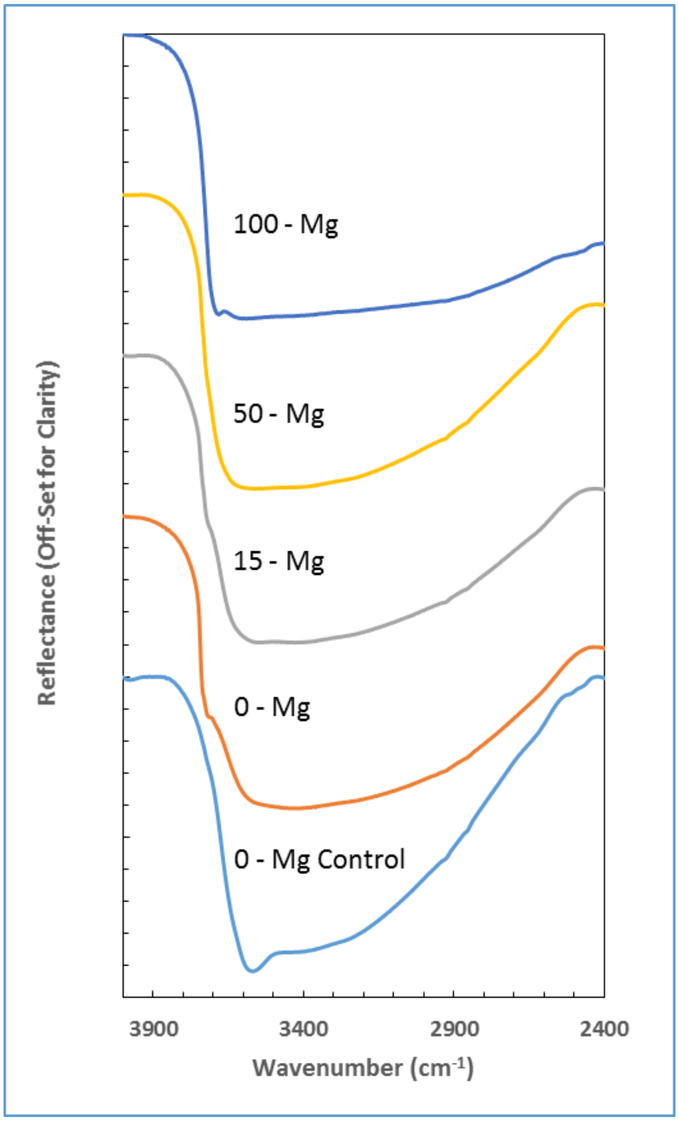

Supplementary Figure 17: Spectra of the 4000 to 2400 cm^{-1} region, for 100° C precipitates. Note the systematic shift of absorptions to higher energies (lower wavenumber) with increasing Mg with the 3Mg-OH absorption occurring at 2.72 μm (3676 cm^{-1}) in Mg-rich clay minerals. The 2Fe-OH absorption occurs at a lower wavelength of 2.80 μm (3571 cm^{-1}). These spectra have absorptions characteristic of Fe and/or Mg-rich clay minerals.

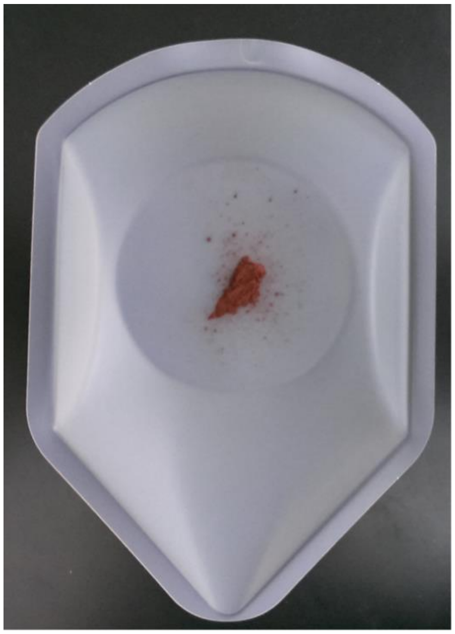

Supplementary Figure 18: Photograph of the 100-Fe^{3+} precipitate (150° C) after drying in-vacuo and grinding to a fine powder in a mortar and pestle. Note the reddish color which is likely from the presence of Fe^{3+}.

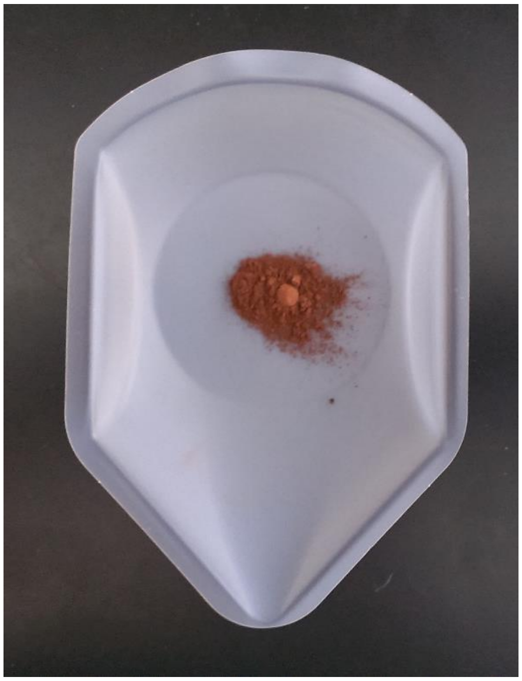

Supplementary Figure 19: Photograph of the 100-Fe^{3+} precipitate (100º C) after drying in-vacuo and grinding to a fine powder in a mortar and pestle. Note the reddish color which is likely from the presence of Fe^{3+}.

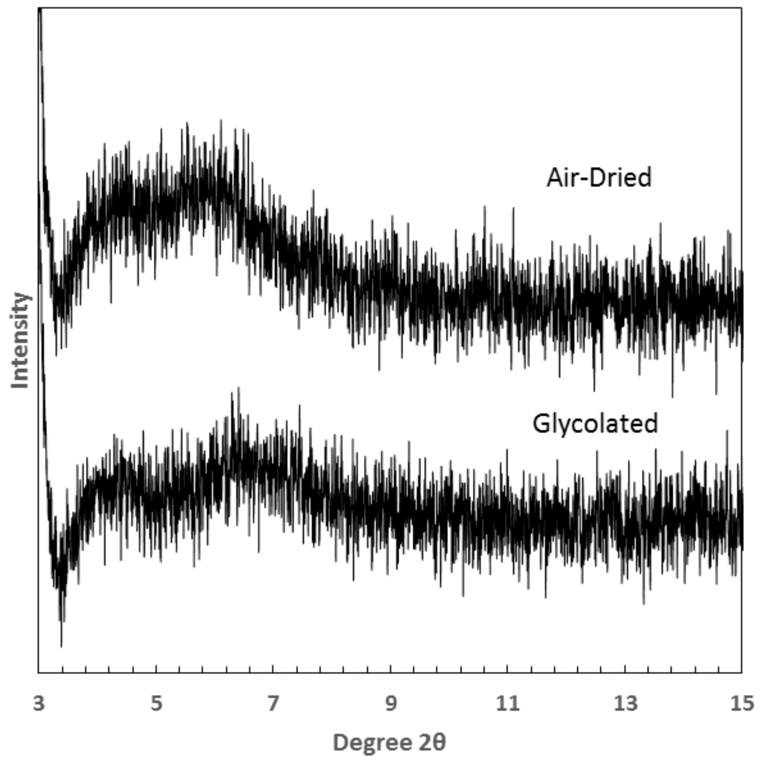

Supplementary Figure 20: X-Ray diffraction pattern of the 100-Fe^{3+} precipitate aged at 150° C - note the lack of crystallinity of this relatively amorphous sample, which indicates the importance of a divalent cation, and is consistent with previous studies[1,2,4]. In addition, no expansion occured when exposing the sample to ethylene glycol vapor for over 24 hours.

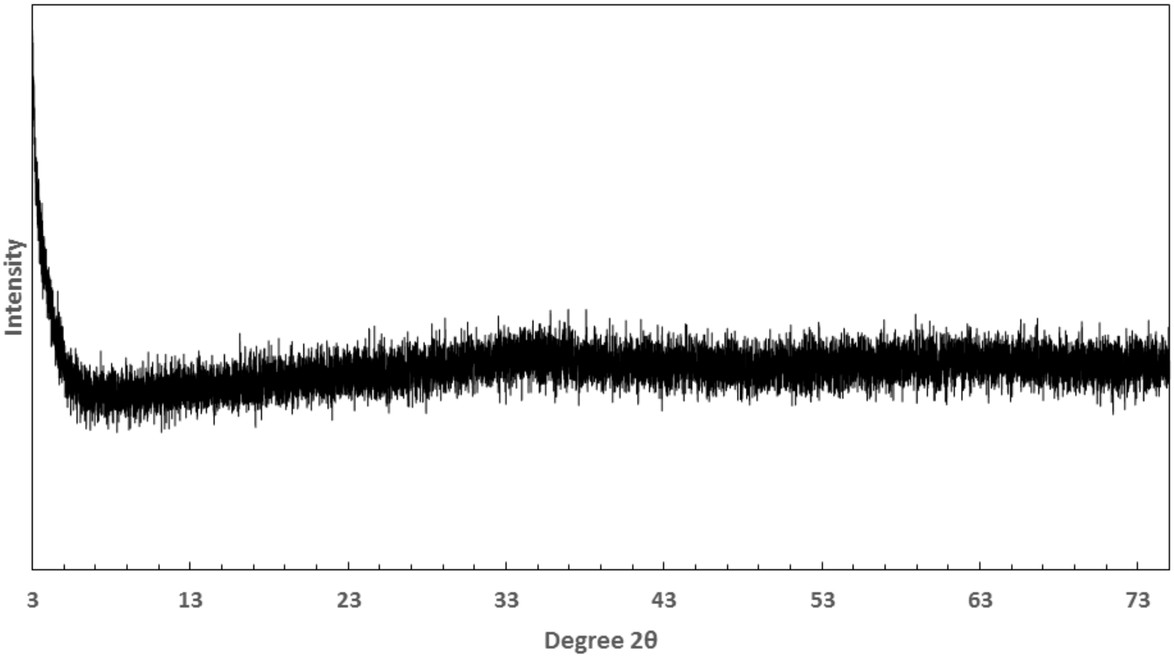

Supplementary Figure 21: X-Ray diffraction pattern of the 100-Fe^{3+} precipitate aged at 100º C - note the lack of crystallinity of this amorphous sample which indicates the importance of a divalent cation, and is consistent with previous studies[1,2,4].

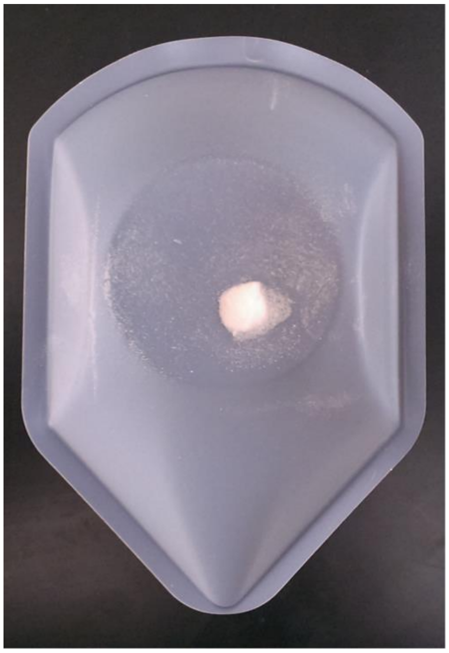

Supplementary Figure 22: Photograph of the 100-Mg precipitate (150º C) after drying in-vacuo and grinding to a fine powder in a mortar and pestle.

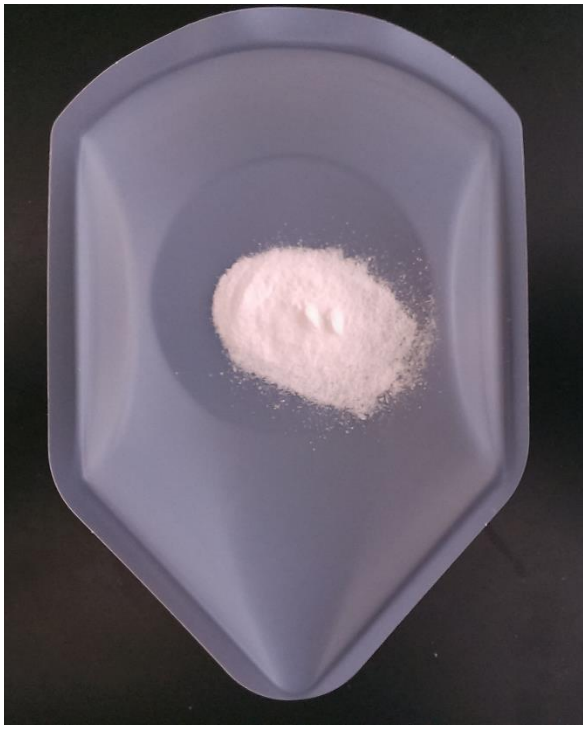

Supplementary Figure 23: Photograph of the 100-Mg precipitate aged at 100º C after drying in-vacuo and grinding to a fine powder in a mortar and pestle.

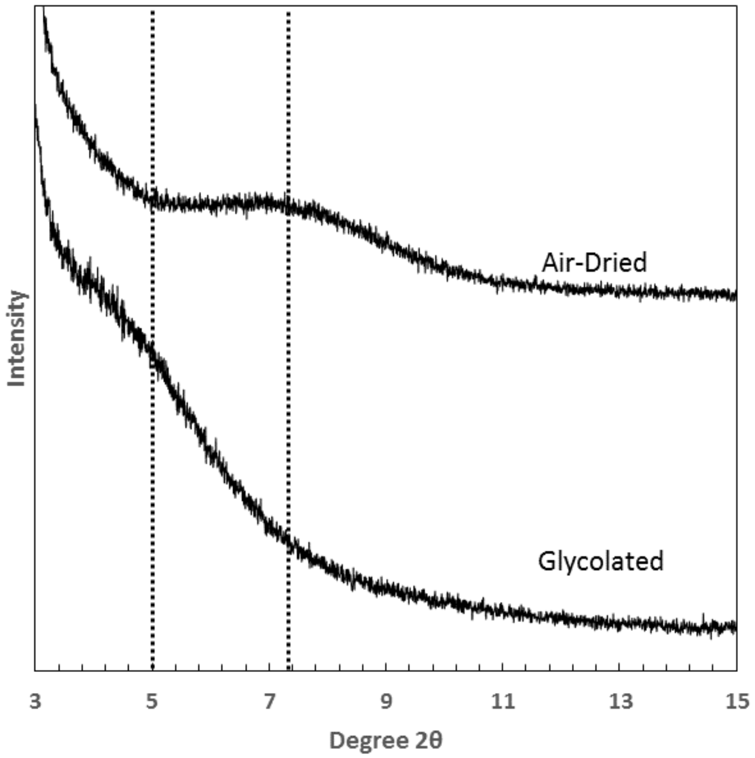

Supplementary Figure 24: X-Ray diffraction pattern of the 100-Mg precipitate aged at 150º C. Note the diffraction peak shifts when exposed to ethylene glycol for over 24 hours. This material lacks crystal coherency in the c-axis which makes it difficult to determine the exact peak positions. This pattern is indicative of a trioctahedral Mg-clay/smectite such as saponite or stevensite.

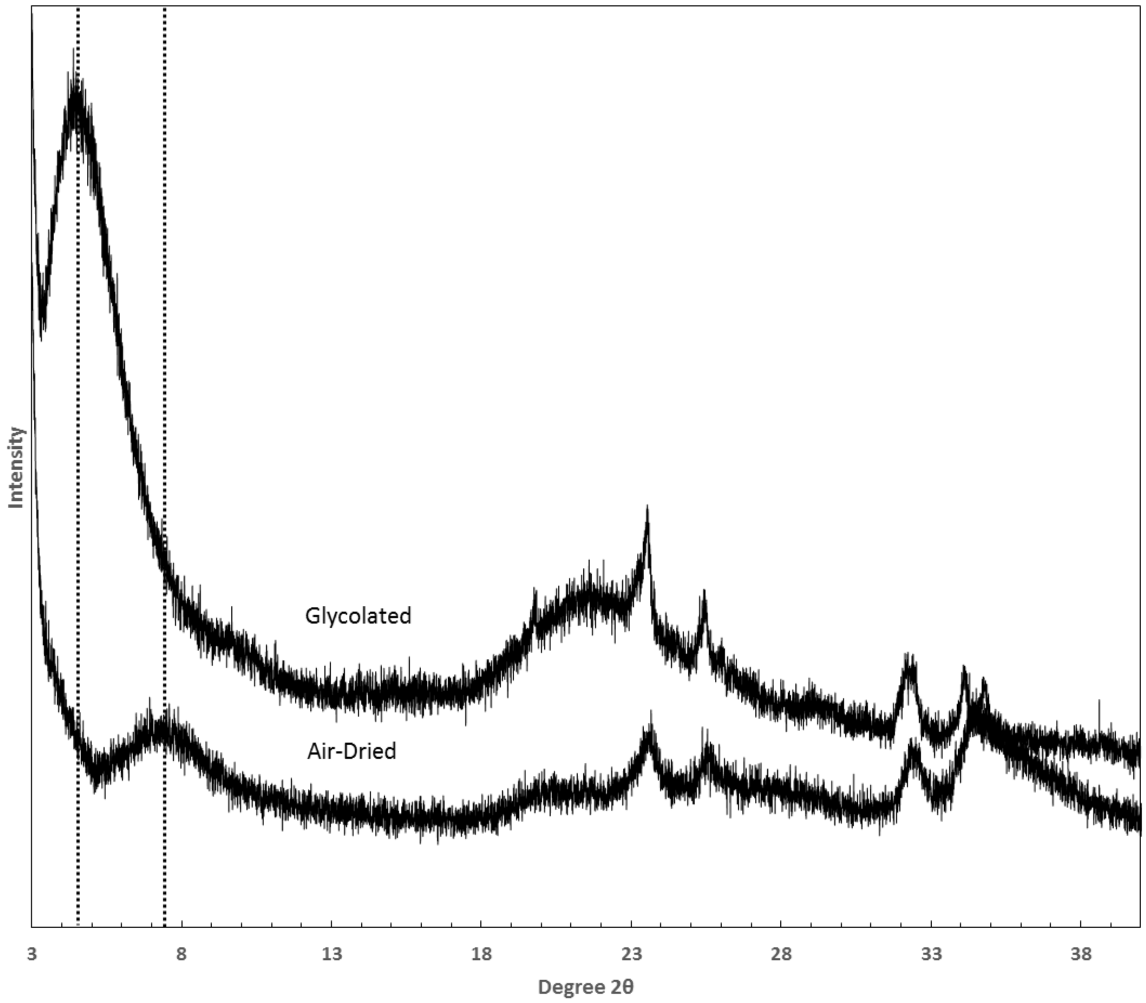

Supplementary Figure 25: X-Ray diffraction pattern of the 100-Mg precipitate aged at 100º C - note the low angle broad reflection, which is indicative of clay minerals. Exposure to ethylene glycol resulted in an expansion of the basal 001 reflection to approximately 20 Å, which is indicative of a smectite. In addition, the broad diffraction occurring at approximately 20 degrees 2-theta characterizes the b-dimension of the unit cell and is used to identify clay mineralogy on Mars[17]. This pattern is indicative of a trioctahedral Mg-smectite such as saponite or stevensite.

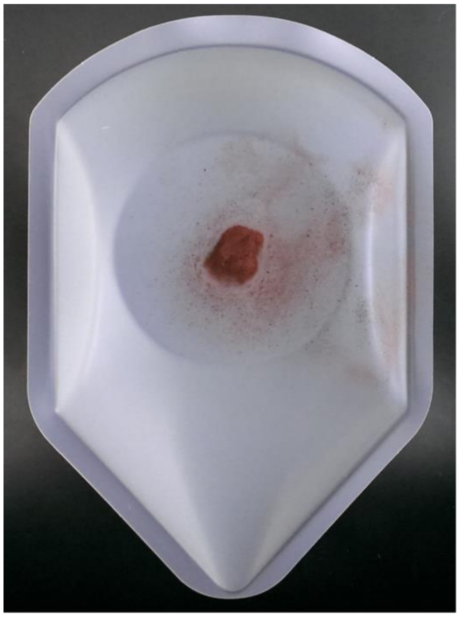

Supplementary Figure 26: Photograph of the 15-Mg 85-Fe^{3+} precipitate (150º C) after drying in-vacuo and grinding to a fine powder in a mortar and pestle.

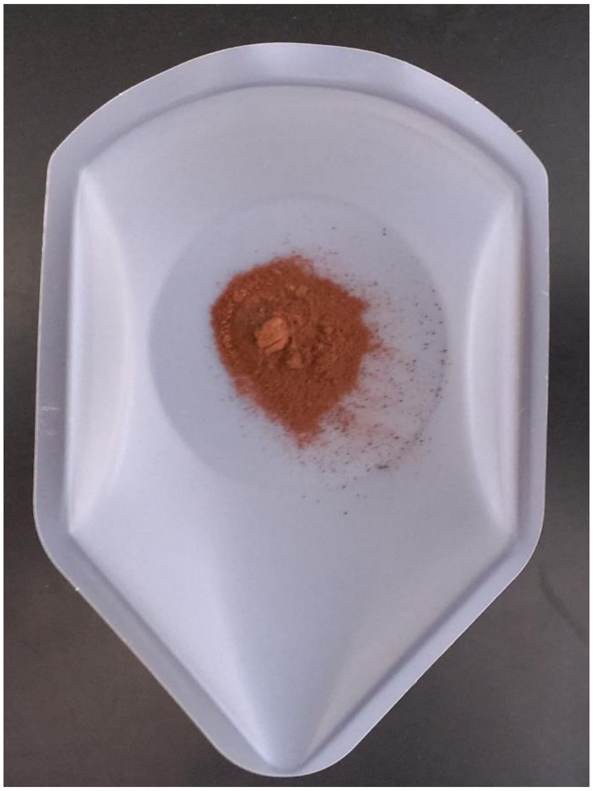

Supplementary Figure 27: Photograph of the 15-Mg 85-Fe^{3+} precipitate (100º C) after drying in-vacuo and grinding to a fine powder in a mortar and pestle.

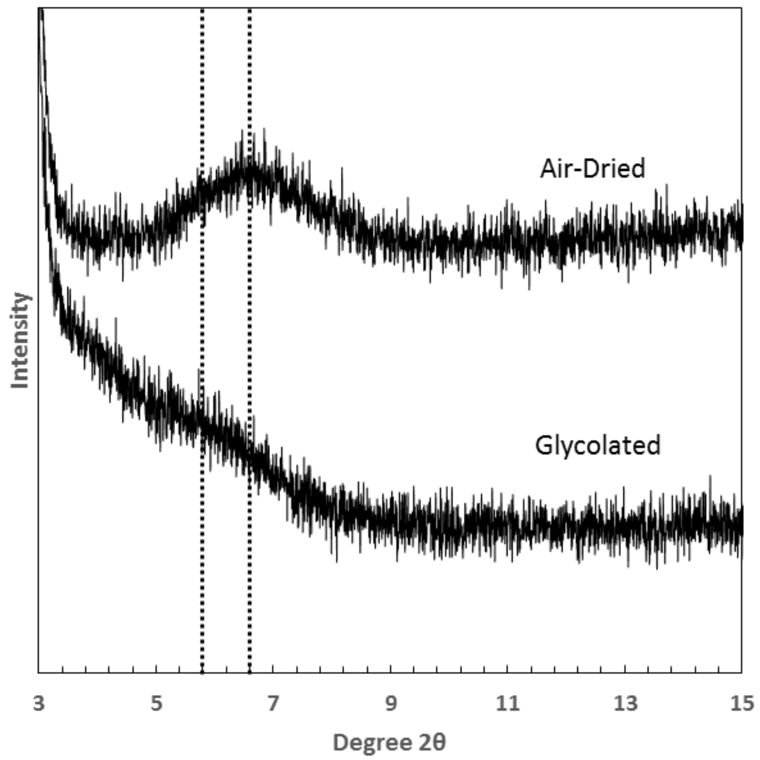

Supplementary Figure 28: X-Ray diffraction pattern of the 15-Mg 85-Fe^{3+} precipitate aged at 150º C. Note the low angle basal reflection under air-dried conditions and the subsequent result when exposed to ethylene glycol vapor for over 24 hours. Due to the loss of the basal reflection resolution, which may be due to the low angle background, it is difficult to determine the exact peak position. However, exposure likely produced an expansion within the clay mineral along the c-axis consistent with a dioctahedral Fe-rich clay mineral.

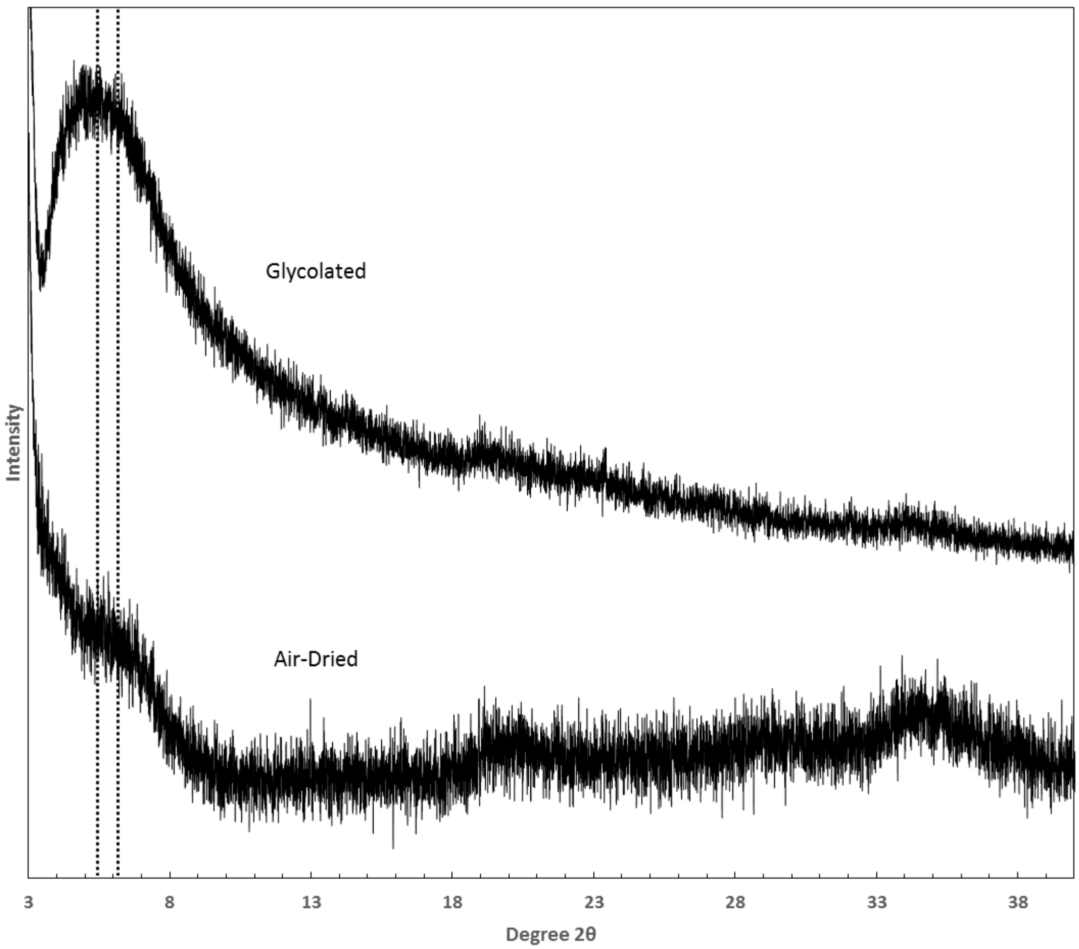

Supplementary Figure 29: X-Ray diffraction pattern of the 15-Mg 85-Fe^{3+} precipitate aged at 100º C – note the low angle reflection (although not prominent, diffraction is occurring at approximately 6 degrees 2θ), which is indicative of clay minerals. In addition, the diffractions occurring at approximately 20 degrees characterizing the b dimension of the unit cell have been used to identify clay mineralogy on Mars[17]. This material is similar to the previously synthesized high-charge nontronites produced by Mizutani et al.[1] and Decarreau et al.[2].

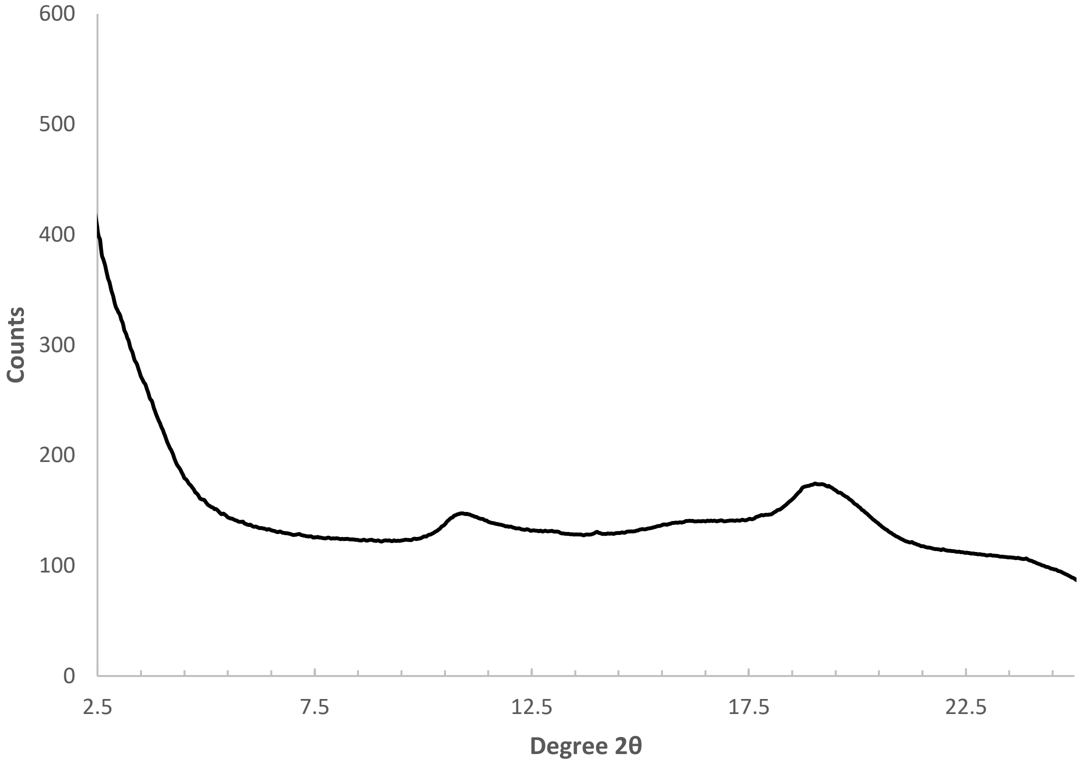

Supplementary Figure 30: Synchrotron μ-XRD (0.86 Å wavelength) pattern for the 15-Mg 85-Fe^{3+} precipitate aged at 150º C. Note the multiple peaks produced due to some crystallinity. This diffraction pattern indicates some clay mineral structure, in that the diffraction peaks are produced through the 020 and 060 reflections.

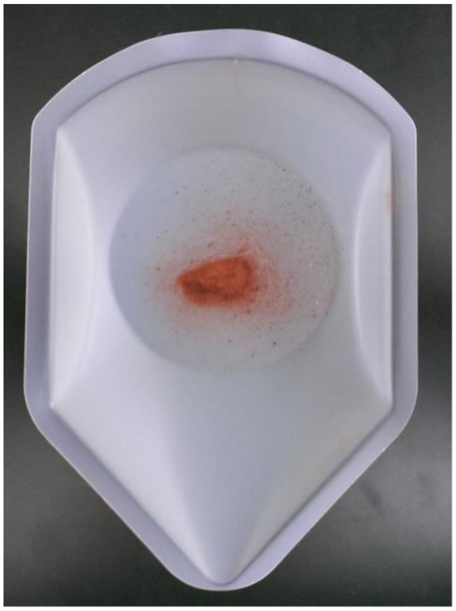

Supplementary Figure 31: Photograph of the 50-Mg 50-Fe^{3+} precipitate (150º C) after drying in-vacuo and grinding to a fine powder in a mortar and pestle.

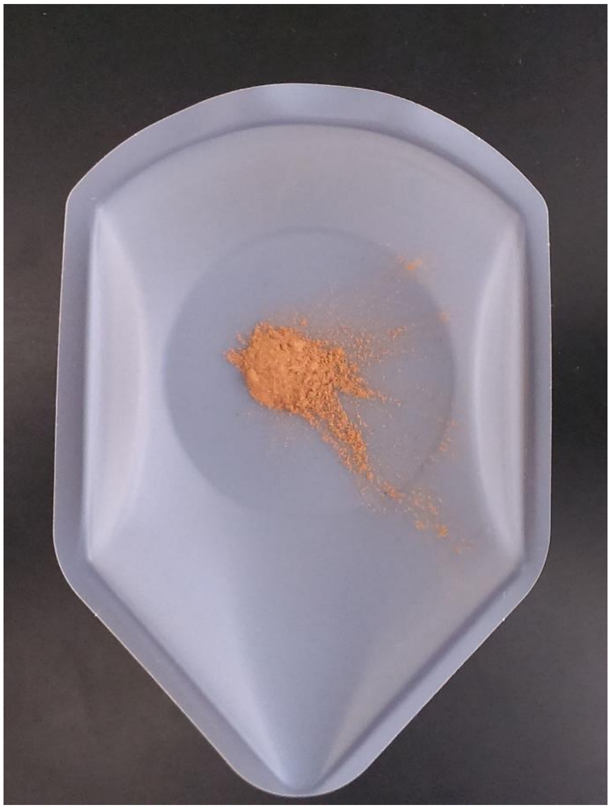

Supplementary Figure 32: Photograph of the 50-Mg 50-Fe^{3+} precipitate (100º C) after drying in-vacuo and grinding to a fine powder in a mortar and pestle.

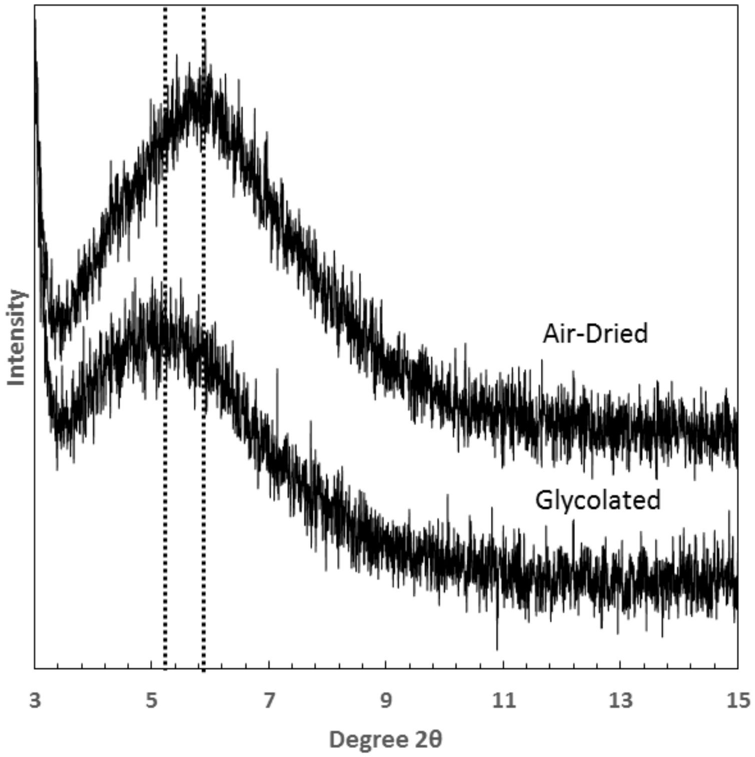

Supplementary Figure 33: X-Ray diffraction pattern of the 50-Mg 50-Fe^{3+} precipitate aged at 150º C. Note the expansion between the air-dried and ethylene glycol treated samples (exposed to vapor for over 24 hours). This expansion to approximately 17 Å is indicative of a smectite clay mineral.

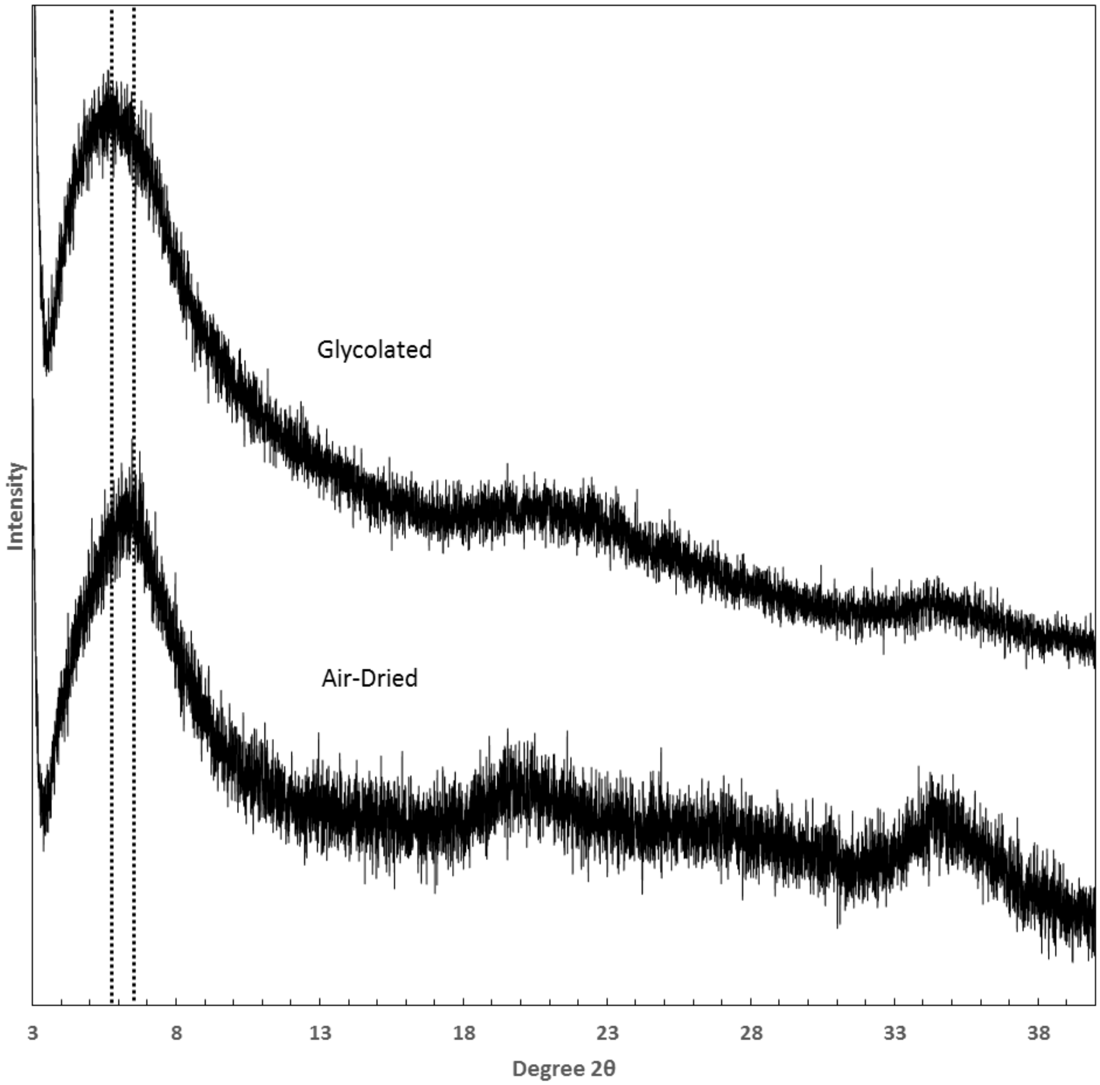

Supplementary Figure 34: X-Ray diffraction pattern of 50-Mg 50-Fe^{3+} precipitate aged at 100° C - note the low angle broad reflection, which is indicative of clay minerals. Exposure to ethylene glycol resulted in a slight expansion from approximately 14 to 15 Å. In addition, the diffractions occurring at approximately 20 degrees characterize the lateral dimension of the b-dimension and have been used to determine clay mineralogy on Mars[17].

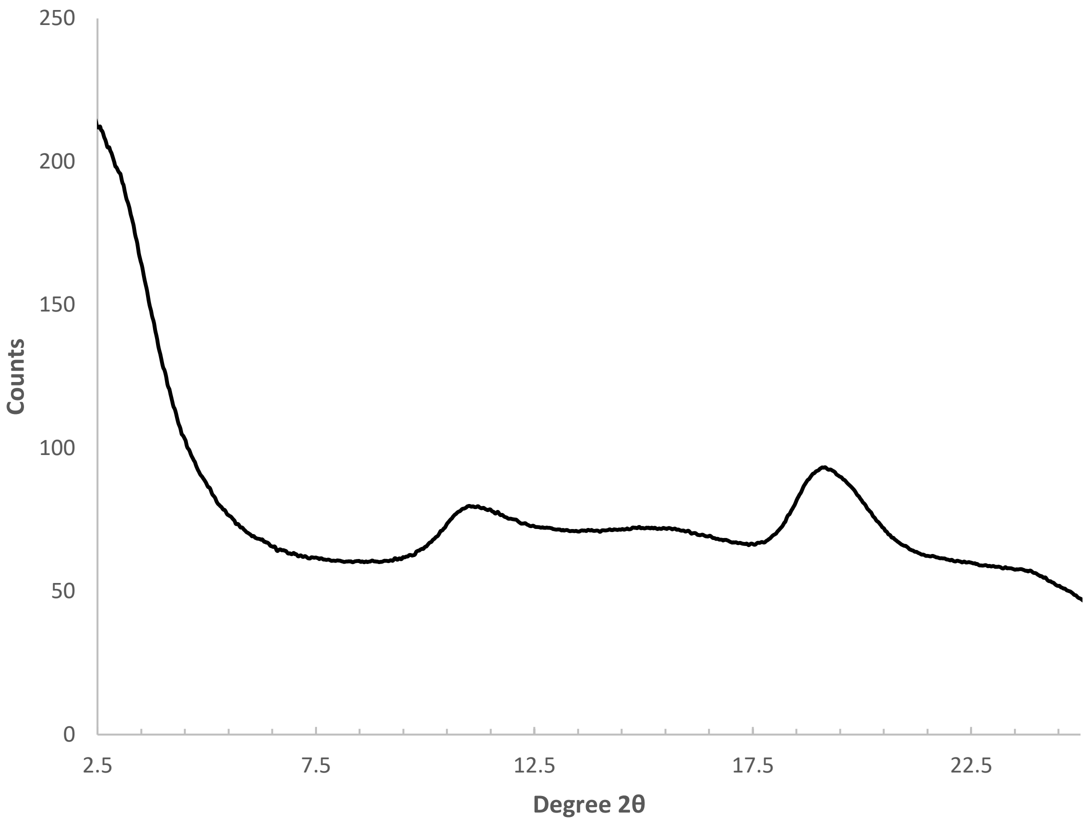

Supplementary Figure 35: Synchrotron μ-XRD (0.86 Å wavelength) pattern for the 50-Mg 50-Fe^{3+} precipitate aged at 150º C. Note the multiple peaks produced due to some crystallinity. This diffraction pattern is consistent with a clay mineral structure in that the diffraction peaks are produced through the 020 and 060 reflections.

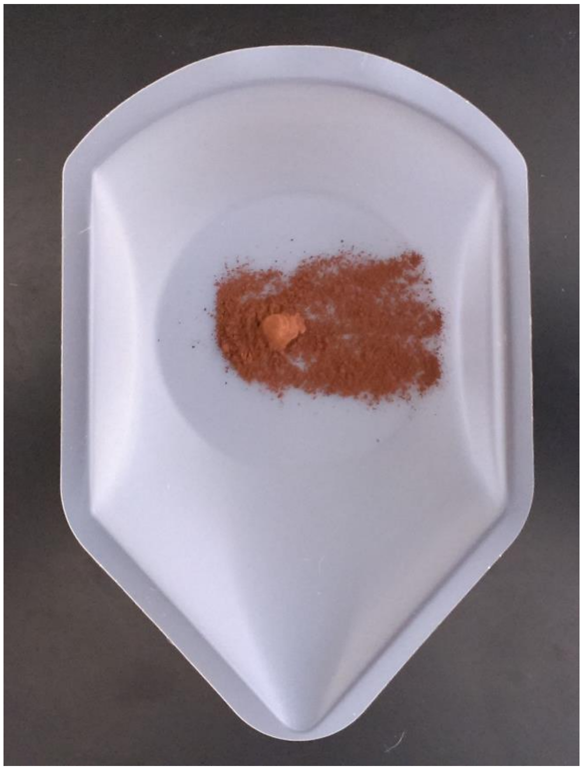

Supplementary Figure 36: Photograph of the 10-Mg 90-Fe^{3+} precipitate at 150º C, after drying in-vacuo and grinding to a fine powder in a mortar and pestle.

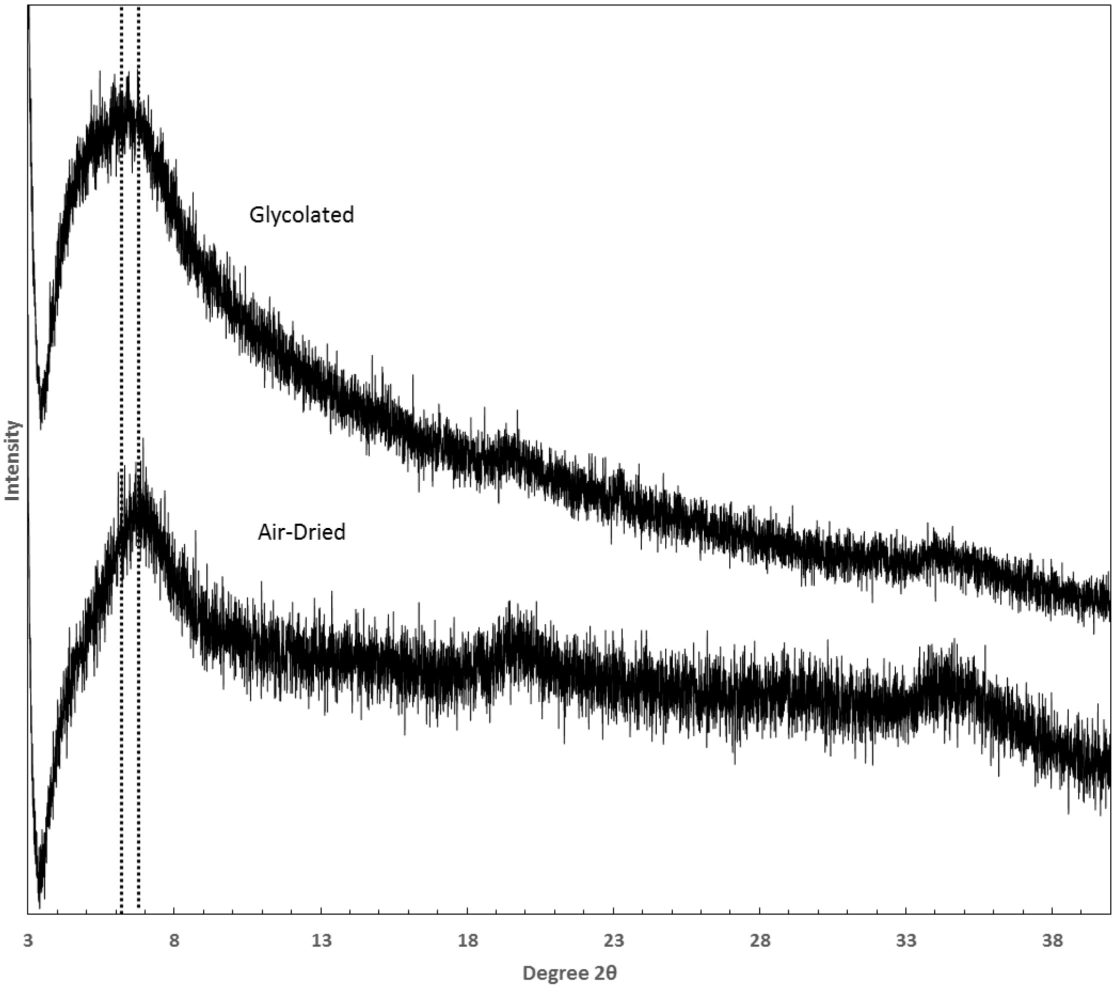

Supplementary Figure 37: X-Ray diffraction pattern of the 10-Mg 90-Fe^{3+} precipitate aged at 150º C. The broad low angle diffraction is indicative of a clay mineral. Note the minimal expansion that occurred between the air-dried and ethylene glycol treated samples (exposed to vapor for over 24 hours). This material is similar to the previously synthesized high-charge nontronites produced by Mizutani et al.[1] and Decarreau et al.[2].

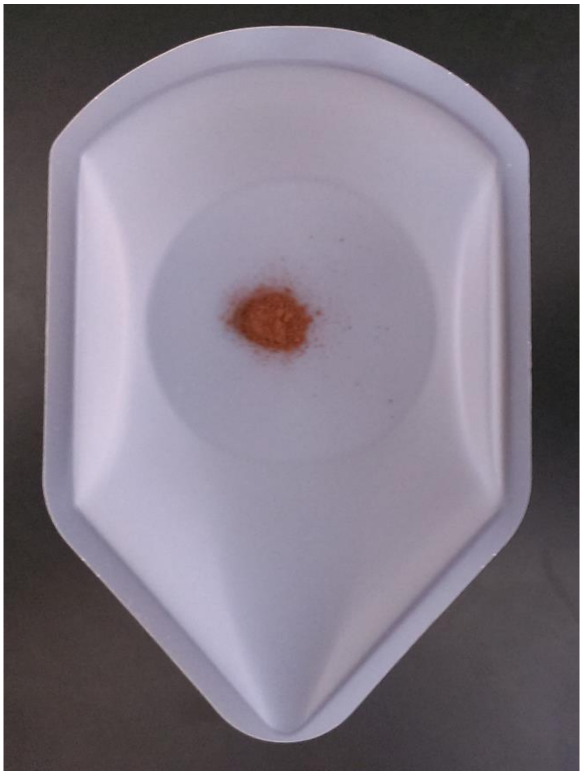

Supplementary Figure 38: Photograph of the 5-Mg 95-Fe^{3+} precipitate aged at 150º C, after drying in-vacuo and grinding to a fine powder in a mortar and pestle.

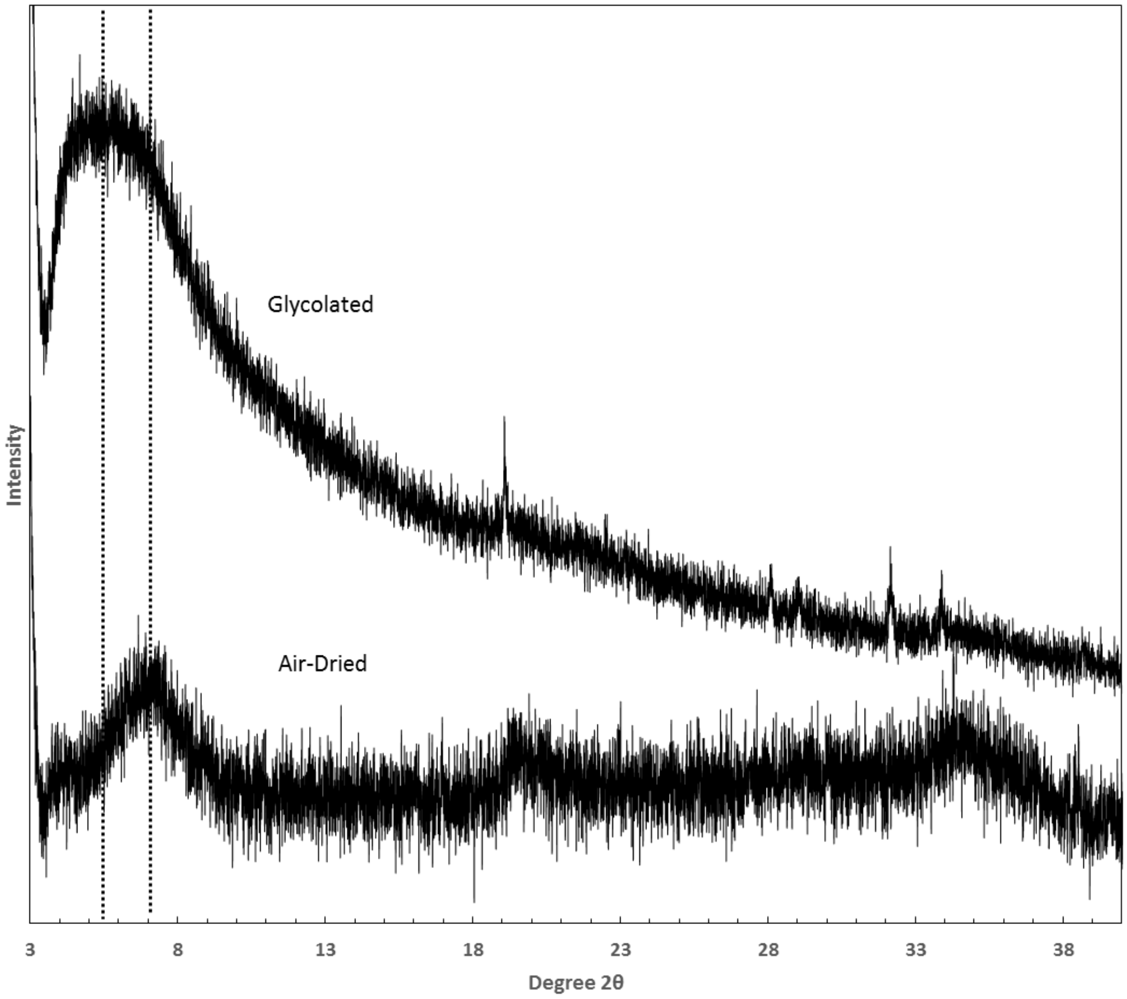

Supplementary Figure 39: X-Ray diffraction pattern of 5-Mg 95-Fe^{3+} precipitated at 150º C - the diffraction pattern of the product is consistent with a poorly crystalline clay mineral. This indicates that Fe-rich clay minerals can precipitate with very minor concentrations of Mg. Note that the expansion which occurred between the air-dried and ethylene glycol treated samples (exposed to vapor for over 24 hours), was less than the characteristic 17 Å of smectite. However, this material is similar to previously studied materials which were characterized as high-charge (e.g. does not fully expand when exposed to ethylene-glycol vapor) nontronites[1,2].

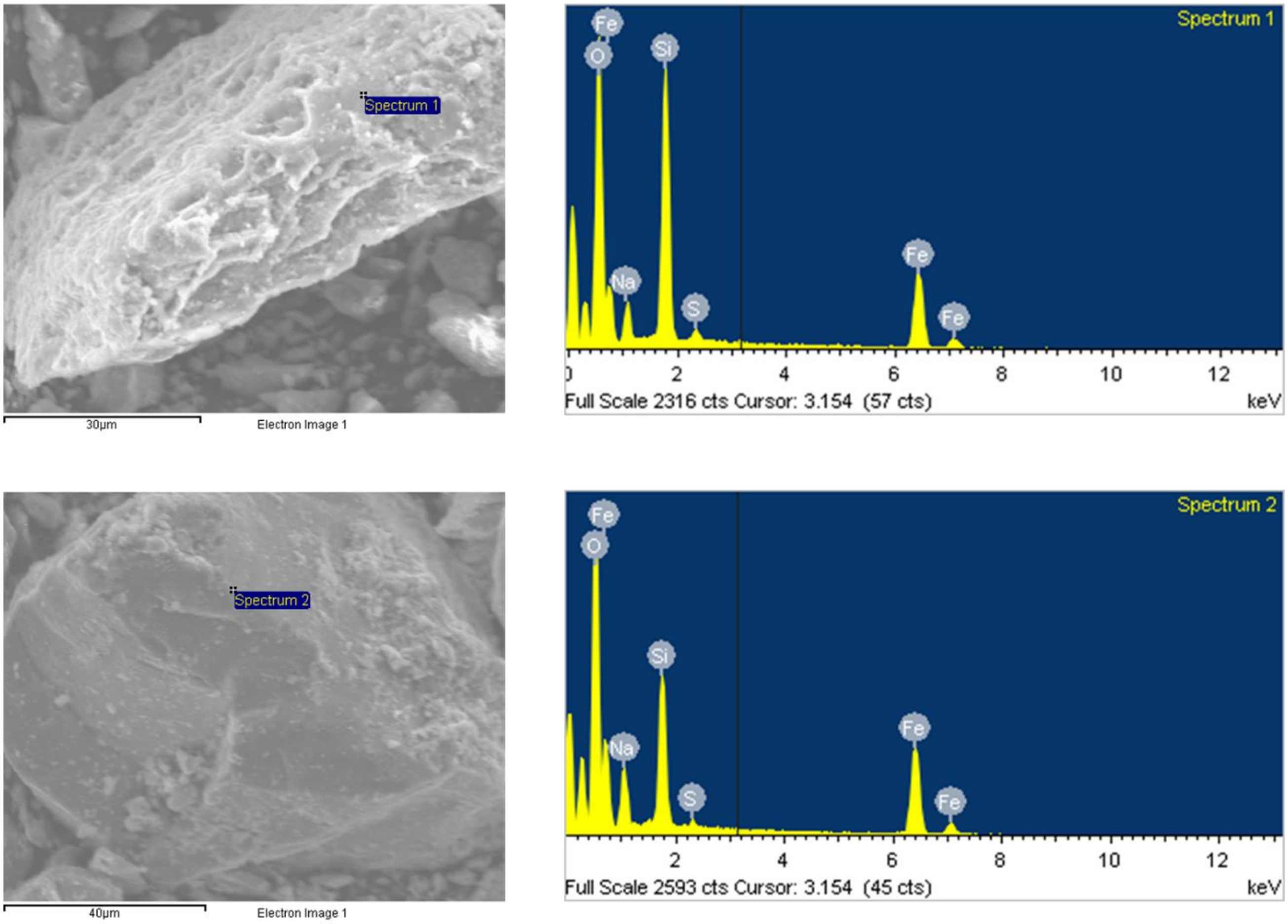

Supplementary Figure 40: EDS spectra and SEM images of 0-Mg 100-Fe control (initially reducing - top), and 0-Mg 100-Fe (oxidized - bottom). Notice the extremely fine grained nature of the precipitates.

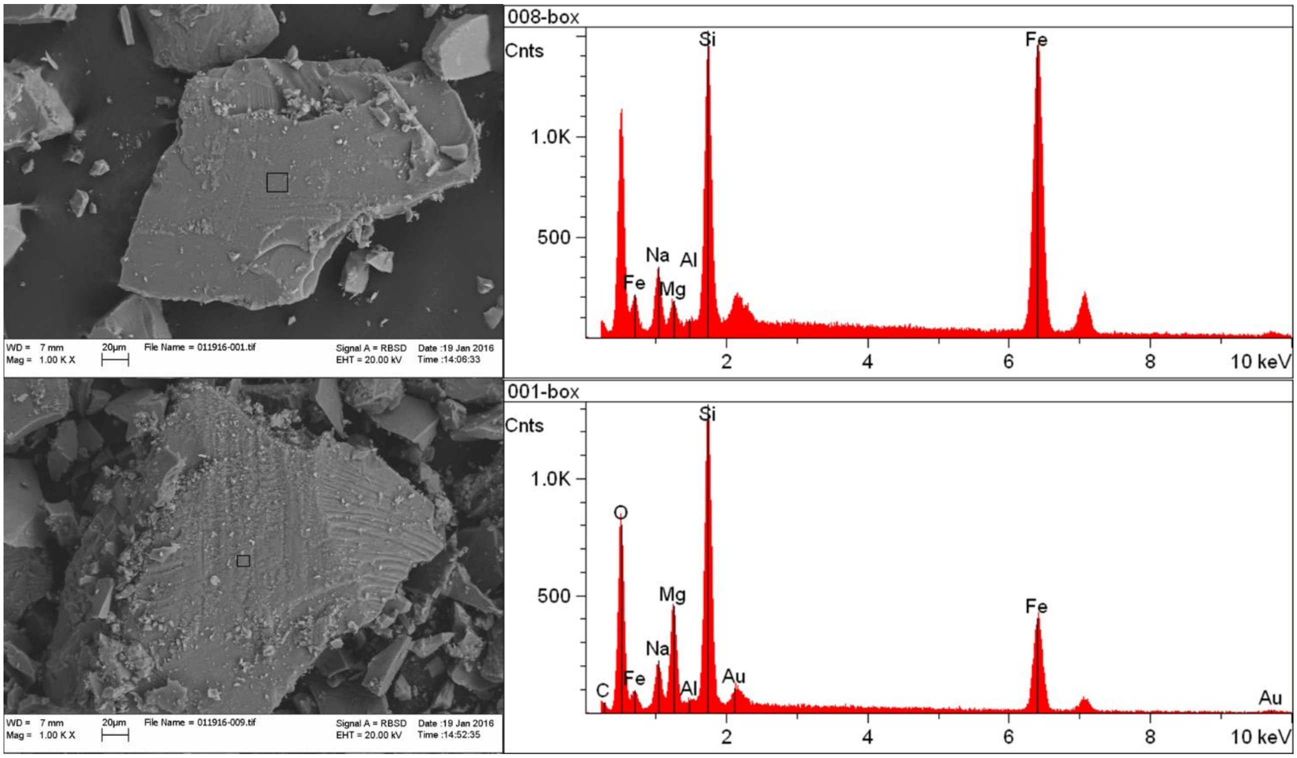

Supplementary Figure 41: EDS spectra and SEM images of the 15-Mg 85-Fe (top), and 5-Mg 95-Fe experiments (bottom). Notice the extremely fine grained nature of the precipitates.

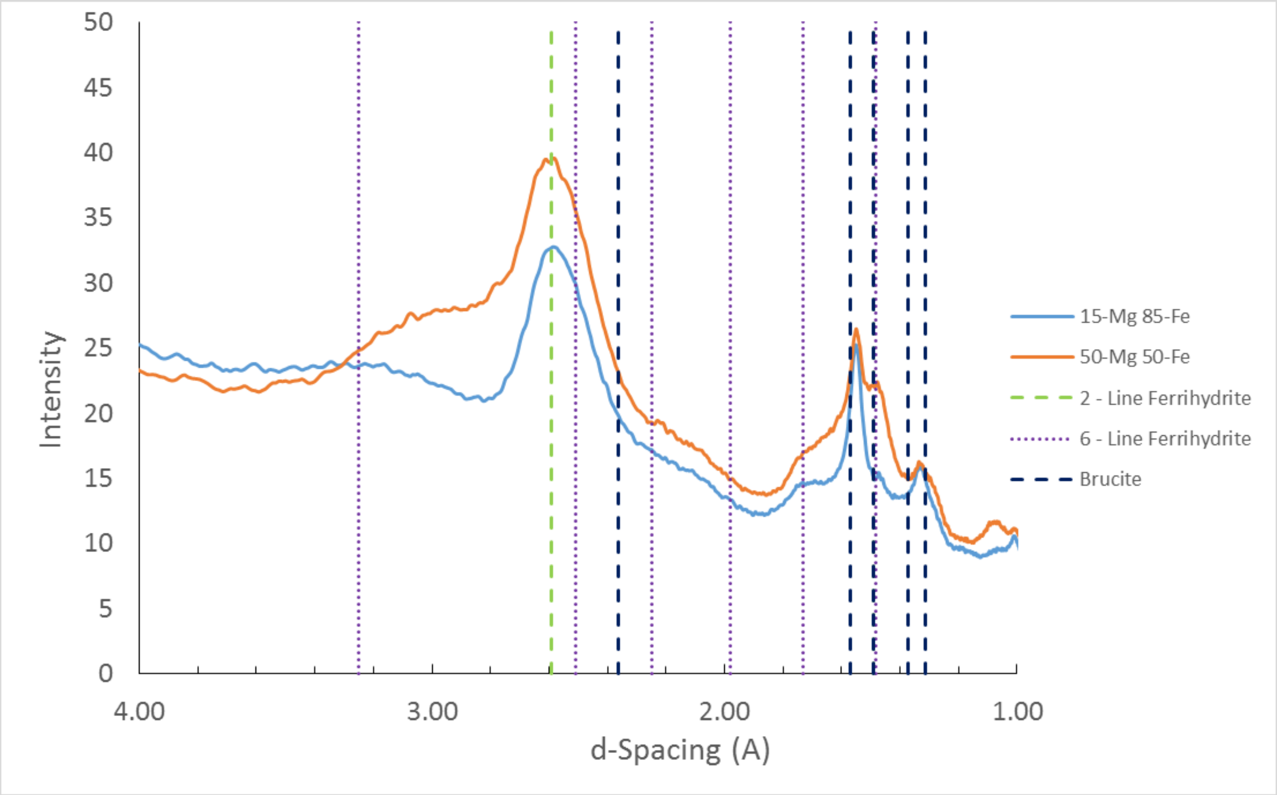

Supplementary Figure 42: Synchrotron µ-XRD patterns of 15-Mg 85-Fe and 50-Mg 50-Fe experiments. Note that both samples show an indication that ferrihydrite may be present within these precipitates. In addition, the higher intensity within the 50-Mg 50-Fe relative to the 15-Mg 85-Fe experiment at approximately 1.5 and 1.25 Å is suggestive of the presence of brucite.

Supplementary References

1 Mizutani, T., Fukushima, Y., Okada, A., Kamigaito, O. & Kobayashi, T. Synthesis of 1: 1 and 2: 1 iron phyllosilicates and characterization of their iron state by Mössbauer spectroscopy. *Clays and Clay Minerals* **39**, 381-386 (1991).

2 Decarreau, A. *et al.* Hydrothermal synthesis, between 75 and 150 C, of high-charge, ferric nontronites. *Clays and Clay minerals* **56**, 322-337 (2008).

3 Badaut, D., Besson, G., Decarreau, A. & Rautureau, R. Occurrence of a ferrous, trioctahedral smectite in recent sediments of Atlantis II Deep, Red Sea. *Clay Minerals* **20**, 389-404 (1985).

4 Harder, H. Nontronite synthesis at low temperatures. *Chemical Geology* **18**, 169-180 (1976).

5 Baldermann, A. *et al.* The Fe-Mg-saponite solid solution series–a hydrothermal synthesis study. *Clay minerals* **49**, 391-415 (2014).

6 Harder, H. Synthesis of iron layer silicate minerals under natural conditions. *Clays Clay Miner* **26**, 65-72 (1978).

7 Poulet, F. *et al.* Phyllosilicates on Mars and implications for early Martian climate. *Nature* **438**, 623-627 (2005).

8 Grauby, O., Petit, S., Decarreau, A. & Baronnet, A. The nontronite-saponite series: an experimental approach. *European Journal of Mineralogy* **6**, 99-112 (1994).

9 Keeling, J. L., Raven, M. D. & Gates, W. P. Geology and characterization of two hydrothermal nontronites from weathered metamorphic rocks at the Uley graphite mine, South Australia. *Clays and Clay Minerals* **48**, 537-548 (2000).

10 Tardy, Y. & Fritz, B. An ideal solid solution model for calculating solubility of clay minerals. *Clay Minerals* **16**, 361-373 (1981).

11 Goodman, B., Russell, J., Fraser, A. & Woodhams, F. A Mössbauer and IR spectroscopic study of the structure of nontronite. *Clays and Clay Minerals* **24**, 53-59 (1976).

12 Manceau, A. *et al.* Oxidation-reduction mechanism of iron in dioctahedral smectites: I. Crystal chemistry of oxidized reference nontronites. *American Mineralogist* **85**, 133-152 (2000).

13 Ribeiro, F. R., Fabris, J. D., Kostka, J. E., Komadel, P. & Stucki, J. W. Comparisons of structural iron reduction in smectites by bacteria and dithionite: II. A variable-temperature Mössbauer spectroscopic study of Garfield nontronite. *Pure and Applied Chemistry* **81**, 1499-1509 (2009).

14 Taylor, G. L., Ruotsala, A. & Keeling Jr, R. Analysis of iron in layer silicates by Mössbauer spectroscopy. *Clays & Clay Minerals* **16**, 381-391 (1968).

15 Vandenberghe, R. E. & De Grave, E. in *Mössbauer Spectroscopy* 91-185 (Springer, 2013).

16 Weldon, R. J., Thomas, W. M., Boslough, M. B. & Ahrens, T. J. Shock-induced color changes in nontronite: implications for the Martian fines. *Journal of Geophysical Research B* **87**, 10102-10114 (1982).

17 Treiman, A. H. *et al.* Ferrian saponite from the Santa Monica Mountains (California, USA, Earth): Characterization as an analog for clay minerals on Mars with application to Yellowknife Bay in Gale Crater. *American Mineralogist* **99**, 2234-2250 (2014).

18 Moore, D. M. & Reynolds, R. C. *X-ray Diffraction and the Identification and Analysis of Clay Minerals*. Vol. 378 (Oxford university press Oxford, 1989).

19 Potts, P., Webb, P. & Watson, J. Energy-dispersive x-ray fluorescence analysis of silicate rocks for major and trace elements. *X-Ray Spectrometry* **13**, 2-15 (1984).

20 Gates, W., Slade, P., Manceau, A. & Lanson, B. Site occupancies by iron in nontronites. *Clays and Clay Minerals* **50**, 223-239 (2002).

21 Sturhahn, W. CONUSS and PHOENIX: Evaluation of nuclear resonant scattering data. *Hyperfine Interactions* **125**, 149-172 (2000).
